# Foliar application of ascorbic acid enhances salinity stress tolerance in barley (*Hordeum vulgare* L.) through modulation of morpho-physio-biochemical attributes, ions uptake, osmo-protectants and stress response genes expression

**DOI:** 10.1016/j.sjbs.2021.03.045

**Published:** 2021-03-21

**Authors:** Amara Hassan, Syeda Fasiha Amjad, Muhammad Hamzah Saleem, Humaira Yasmin, Muhammad Imran, Muhammad Riaz, Qurban Ali, Faiz Ahmad Joyia, Mobeen, Shakeel Ahmed, Shafaqat Ali, Abdulaziz Abdullah Alsahli, Mohammed Nasser Alyemeni

**Affiliations:** a Department of Botany, Government College University, Allama Iqbal Road, 38000 Faisalabad, Pakistan; b Department of Botany University of Agriculture Faisalabad, Punjab, Pakistan; c MOA Key Laboratory of Crop Ecophysiology and Farming System in the Middle Reaches of the Yangtze River, College of Plant Science and Technology, Huazhong Agricultural University, Wuhan 430070, China; d Department of Bio-Sciences, COMSATS University, Islamabad 45550, Pakistan; e Department of Crop Science and Technology, College of Agriculture, South China Agricultural University, Guangzhou 510642, Guangdong, China; f Root Biology Center, College of Natural Resources and Environment, South China Agricultural University, Guangzhou 510642, Guangdong, China; g Key Laboratory of Plant Pathology, College of Plant Science & Technology, Huazhong Agricultural University, Wuhan 430070, China; h Centre of Agricultural Biochemistry and Biotechnology (CABB), University of Agriculture Faisalabad; i Instituto de Farmacia, Facultad de Ciencias, Universidad Austral de Chile, Valdivia 5110566, Chile; j Department of Environmental Sciences and Engineering, Government College University Allama Iqbal Road, 38000 Faisalabad, Pakistan; k Department of Biological Sciences and Technology, China Medical University, Taichung 40402, Taiwan; l Department of Botany and Microbiology, College of Science, King Saud University, 11451-Riyadh, Saudi Arabia

**Keywords:** Antioxidant response, Cereal crop, Oxidative stress, Relative gene expression, Salt stress, Stomatal properties

## Abstract

Barley (*Hordeum vulgare* L.) is a major cereal grain and is known as a halophyte (a halophyte is a salt-tolerant plant that grows in soil or waters of high salinity). We therefore conducted a pot experiment to explore plant growth and biomass, photosynthetic pigments, gas exchange attributes, stomatal properties, oxidative stress and antioxidant response and their associated gene expression and absorption of ions in *H. Vulgare*. The soil used for this analysis was artificially spiked at different salinity concentrations (0, 50, 100 and 150 mM) and different levels of ascorbic acid (AsA) were supplied to plants (0, 30 and 60 mM) shortly after germination of the seed. The results of the present study showed that plant growth and biomass, photosynthetic pigments, gas exchange parameters, stomatal properties and ion uptake were significantly (*p* < 0.05) reduced by salinity stress, whereas oxidative stress was induced in plants by generating the concentration of reactive oxygen species (ROS) in plant cells/tissues compared to plants grown in the control treatment. Initially, the activity of antioxidant enzymes and relative gene expression increased to a saline level of 100 mM, and then decreased significantly (*P* < 0.05) by increasing the saline level (150 mM) in the soil compared to plants grown at 0 mM of salinity. We also elucidated that negative impact of salt stress in *H. vulgare* plants can overcome by the exogenous application of AsA, which not only increased morpho-physiological traits but decreased oxidative stress in the plants by increasing activities of enzymatic antioxidants. We have also explained the negative effect of salt stress on *H. vulgare* can decrease by exogenous application of AsA, which not only improved morpho-physiological characteristics, ions accumulation in the roots and shoots of the plants, but decreased oxidative stress in plants by increasing antioxidant compounds (enzymatic and non-enzymatic). Taken together, recognizing AsA's role in nutrient uptake introduces new possibilities for agricultural use of this compound and provides a valuable basis for improving plant tolerance and adaptability to potential salinity stress adjustment.

## 1 Introduction

Stress is characterized as any external limitation of abiotic (salinity, heat, water, etc.) or biotic (herbivore) that limits the photosynthesis rate and decreases the ability of a plant to convert energy into biomass (Alam et al., 2020, Ali et al., 2020, Nazar et al., 2020, Zaheer et al., 2020b). World agriculture faces many problems, such as the production of 70% more food for the increasing population, and crop productivity does not increase in tandem with food demand (Ali et al., 2021, Parihar et al., 2015, Saleem et al., 2020b, Zaheer et al., 2020d). In certain situations, lower productivity is due to various abiotic stresses. The reduction of crop losses due to different environmental stressor is a major area of concern to cope with the growing requirements for food (Deng et al., 2021, Hassini et al., 2017, Javed et al., 2020, Jiang et al., 2016, Zaheer et al., 2020a). The main abiotic stresses such as high salinity, drought, cold and heat have a negative effect on the survival, development of biomass and yield of staple food crops of up to 70% (Gupta and Thind, 2017, Hashmat et al., 2021, Yaseen et al., 2020, Zaheer et al., 2020d). Among these abiotic stresses, much of the crop research carried out due to global economic losses, estimated at around $14–19 million US dollars, has centered on soil salinity (Jing et al., 2018, Munns and Tester, 2008, Zafar et al., 2015). Salt stress mainly affects plants in two ways, i.e., osmotic stress, induced by the deposition of large quantities of soluble salts in the soil, which reduces the supply of soil water to plants; and ion toxicity, caused by high salt accumulation within the plant, which disrupts a number of metabolic processes, including the inactivation of certain enzymes (Kaya et al., 2015, Parida and Das, 2005, Safdar et al., 2019, Zamin and Khattak, 2017). Salinization is rapidly accelerating, occupying about 20% of the total irrigated area of the world and resulting in a 50% loss of arable land by 2050, especially in most fertile areas of the world, i.e., the Mediterranean region and South Asia (Alam et al., 2020, Bolat et al., 2006, Munns and Tester, 2008, Sadak and Abdelhamid, 2015). Soil salinity has been shown to exist long before humans and agriculture, but agricultural activities such as irrigation have increased the issue (Iqbal et al., 2015, Rahneshan et al., 2018). In the growth medium, high salinity concentration imposes strong deleterious effects on plant biomass (Li et al., 2020), physiology (Nizam, 2011) accumulation of mineral ions (Liu et al., 2020), destroy PSII reactions (Khan et al., 2013), and biochemical damage due to production of reactive oxygen species (ROS) which eventually leads to poor growth and metabolic damage of the plant (Zafar et al., 2015). The disruption of the balance between the production of ROS and antioxidant defense systems that triggers excessive accumulation of ROS and induces oxidative stress in plants is one of the most significant consequences of salt stress (Saleem et al., 2020a, Saleem et al., 2020f, Saleem et al., 2020h). Notably, both enzymatic and non-enzymatic antioxidant defense systems under extreme salinity stresses maintain the balance between detoxification and ROS generation (Islam et al., 2016, Kala, 2015, Shafiq et al., 2020). These ROS include: hydrogen peroxide (H_2_O_2_), singlet oxygen (^1/2^O_2_), superoxide anion (O_2_^•–^), hydroxyl (HO^•^), alkoxyl (RO^•^), peroxyl (RO_2_^•^), and organic hydroperoxide (ROOH) and can scavenged by varieties of antioxidants such as superoxide dismutase (SOD), peroxidase (POD) and catalase (CAT) and ascorbate peroxidase (APX) (Javed et al., 2021, Rehman et al., 2019, Zaheer et al., 2020c).

Ascorbic acid (AsA), also referred to as vitamin C, is a major non-enzyme antioxidant in plants and plays an important role in mediating certain oxidative stresses caused by biotic and abiotic stress (Akhlaghi et al., 2018, Sharma et al., 2019). AsA can enhance the growth of a plant and boost its capacity to withstand stress and hydroxyproline-containing proteins must be synthesized (Bilska et al., 2019). Moreover, AsA is the first line of plant defense against oxidative stress by removing a number of free radicals, such as O_2_^•–^, HO^•^, and H_2_O_2_, mostly as a substrate of APX, an essential enzyme of the ascorbate–glutathione pathway (Bilska et al., 2019, Sharma et al., 2019). Ascorbate is a cofactor for several cellular enzymes, such as violaxanthin de-epoxidase, which is essential for photoprotection by xanthophyll cycle and other enzymes and is directly involved in the removal of ROS, and the addition of exogenous AsA will inhibit lipid peroxidation and decrease malondialdehyde (MDA) content in plant tissues, thus improving the antioxidant ability of plant tissues (Munir and Aftab, 2011, Zhou et al., 2016). The effect of ascorbic acid on improving the salinity tolerance of potatoes was studied by Sajid and Aftab (Sajid and Aftab, 2009). They noted that the activity of most antioxidant enzymes, such as SOD, POD, CAT and APX, increased significantly under NaCl stress conditions after exogenous application of ascorbic acid, thereby improving plant survival under overall environmental stress. Younis et al. (Younis et al., 2010) also stated that a marked and statistically significant increase in the percentage resistance to salt stress and the growth of *Vicia faba* seedlings was caused by the exogenous addition of 4 mM ascorbic acid with NaCl to the stressful media during the duration of the experiment (12 days). In their analysis, Aly et al. (Aly et al., 2012) observed that the addition of 1 mM of ascorbic acid to Egyptian clover (*Trifolium alexandrinum* L.) seedlings grown in NaCl medium significantly increased the germination of seeds, the content of carotenoids and chlorophyll and the dry mass of seedlings grown in NaCl medium.

*Hordeum vulgare* L. (Barley), among the crops is one of the most economically important plants, considered an outstanding model for agronomy, plant physiology and abiotic stress studies, with a rapid growth rate and high adaptability to different environments (Hossain and Akhtar, 2014, Ullah et al., 2016). It is an important cereal, cultivated mainly for animal feed and as a raw material for the manufacture of alcohol. Moreover, the production of *H. vulgare* in Asia is affected by growing dryland salinity, which significantly limits growth and decreases yields (Abdel-Hamid and Mohamed, 2014, Agami, 2014). It is noteworthy, however, because it can sustain growth despite accumulating high salt concentrations in its tissues. Sequestration of NaCl into intracellular vacuoles and the synthesis of compatible solutes accumulating in the cytoplasm are likely to require the high tissue tolerance of *H. vulgare* to balance the osmotic ability of the vacuolar NaCl (Ali et al., 2011, Ullah et al., 2016). Therefore, *H. vulgare* is a plant of particular interest for metabolomic studies with the longer-term objective of passing the tissue tolerance trait to other commercial plants with much lower NaCl tissue tolerance, such as *Triticum aestivum* and *Oryza sativa*. While salinity is a serious global problem, cereal crops such as *H. vulgare* are especially important for its production. Numerous studies have been performed by scientists (Abdel-Hamid and Mohamed, 2014, Agami, 2014, Wu et al., 2013), using *H. vulgare* in salt-stressed conditions as a cereal crop and using different methods to mitigate its negative effect. However, minimal studies have been investigated using AsA's foliar application to study various morpho-physiological characteristics and nutrient uptake in different parts of plants in salinity soil. We therefore conducted the present study to investigate different plant growth and biomass, photosynthetic pigments, gas exchange attributes, stomatal properties, oxidative stress and antioxidant response and their associated gene expression and absorption of ions in *H. vulgare* in different applications of AsA in salt-stressed environments. The results of this study will contribute to our understanding of (i) the role of various AsA foliar levels on plant growth and biomass, photosynthetic pigments, attributes of gas exchange and stomatal properties, (ii) the oxidative stress and response of antioxidative enzymes and their relative expression of genes, and (iii) the nutritional status of various parts of *H. vulgare* grown under different salinity in the soil. This is, to the best of our knowledge, one of the few studies focusing on plant growth and composition through foliar application of AsA in *H. vulgare* under different salinity levels.

## 2 Materials and methods

### 2.1 Plant material and experimental treatments

A pot experiment was conducted in the University of Agriculture, Faisalabad 38040, Pakistan. Healthy and mature seeds of *H. vulgare* used for the pot experiment were collected from the Ayub Agricultural Institute Faisalabad (AARI), Pakistan. Seeds were surface sterilized with 0.1% HgCl_2_, followed by distilled water for the prevention of surface fungal/bacterial contamination. The non-saline soil was collected from experimental stations of University of Agriculture, Faisalabad and filled in the pots (20-cm-tall, 30-cm-wide). Each pot contains 10 kg of soil and physico-chemical properties of non-saline soil are presented in Table 1S. The non-saline soil was artificially applied at various concentrations of sodium chloride (0, 50, 100 and 150 mM) using sodium chloride (NaCl) salt. NaCl salt was added in the non-saline soil after one week of seed sowing. After 14 days of salinity stress, all seedlings were also supplied by the application (foliar) of AsA at various concentrations (0, 30 and 60 mM). All the spray formulations contained Tween 20 as surfactant. The control plants were mock sprayed with only Tween 20 dissolved in distilled water. AsA were supplied to every parts of the plants including leaves and stems but excluding the roots of the plants. All the plants were watered regularly and different intercultural operations such as weeding were conducted when needed. Recommended fertilization and management practices were followed. The experiment was conducted in the winter of 2015 with completely randomized design (CRD) with five replications of each treatment. All plants were looked after carefully and botanical garden can be covered with plastic sheet, when there were chances of any rainfall. Moreover, in the winter, plant was placed in the open-air botanical garden, where they received natural light with day/night temperatures of 25/15 °C and day/night humidity of 60/70%. The details of the treatments used in this study are as follow: (1) (salinity 0 mM + AsA 0 mM), (2) (salinity 0 mM + AsA 30 mM), (3) (salinity 0 mM + AsA 60 mM), (4) (salinity 50 mM + AsA 0 mM), (5) (salinity 50 mM + AsA 30 mM), (6) (salinity 50 mM + AsA 60 mM), (7) (salinity 100 mM + AsA 0 mM), (8) (salinity 100 mM + AsA 30 mM), (9) (salinity 100 mM + AsA 60 mM), (10) (salinity 150 mM + AsA 0 mM), (11) (salinity 150 mM + AsA 30 mM) and (12) (salinity 150 mM + AsA 60 mM).

### 2.2 Sampling and data collection

All plants were harvested to study different characteristics after 45 days of AsA supplied (precisely 65 days after staring the experiment). Randomly selected plants (from each treatments) were used for data sampling for different physiological traits. For this purpose, a fully expanded leaf was taken (fifth from the top) from the plant in early morning (8:00–9:00 AM). The selected leaves were washed with distilled water and immediately placed in liquid nitrogen and stored at refrigerator (-80 °C). All the plants were rooted-up in the late winter (February) 2016 to study the effect of salinity and AsA on the plants. Grain and plant fresh weight were measured using a digital weighting balance machine. Although, plant height was measured using a measuring tape (from root to shoot tips). However, plant samples were also used to measured dry biomass of the plants, by put it in oven for 72 h at 65 °C and weight was also measured using digital weighting balance, until it become constant.

### 2.3 Determination of photosynthetic pigments and gas exchange parameters

Leaves were collected for determination of chlorophyll and carotenoid contents. For chlorophylls, 0.1 g of fresh leaf sample was extracted with 8 mL of 95% acetone for 24 h at 4 °C in the dark. The absorbance was measured by a spectrophotometer (UV-2550; Shimadzu, Kyoto, Japan) at 646.6, 663.6 and 450 nm. Chlorophyll content was calculated by the standard method of (Arnon, 1949). Stomata were counted at random in 30 visual sections on the abaxial epidermis, and final tallies were used to calculate stomatal density. We used Image J software for measuring stomatal lengths, density, widths, and apertures.

Gas exchange parameters were also measured during the same days. Net photosynthesis (*Pn*), leaf stomatal conductance *(Gs)*, transpiration rate (*Ts*), and intercellular carbon dioxide concentration (*Ci*) were measured from three different plants in each treatment group. Measurements were conducted between 11:30 and 13:30 on days with clear sky. Rates of leaf *Pn*, *Gs, Ts*, and *Ci* were measured with a LI-COR gas-exchange system (LI-6400; LI-COR Biosciences, Lincoln, NE, USA) with a red-blue LED light source on the leaf chamber. In the LI-COR cuvette, CO_2_ concentration was set as 380 mmol mol^−1^ and LED light intensity was set at 1000 mmol m^−2^ s^−1^, which is the average saturation intensity for photosynthesis in *H. vulgare* (Austin, 1990).

### 2.4 Determination of oxidative stress indicators

The degree of lipid peroxidation was evaluated as malondialdehyde (MDA) contents. Briefly, 0.1 g of frozen leaves were ground at 4 °C in a mortar with 25 mL of 50 mM phosphate buffer solution (pH 7.8) containing 1% polyethene pyrrole. The homogenate was centrifuged at 10,000 × *g* at 4 °C for 15 min. The mixtures were heated at 100 °C for 15–30 min and then quickly cooled in an ice bath. The absorbance of the supernatant was recorded by using a spectrophotometer (xMark™ Microplate Absorbance Spectrophotometer; Bio-Rad, United States) at wavelengths of 532, 600 and 450 nm. Lipid peroxidation was expressed as l mol g^−1^ by using the formula: 6.45 (A532-A600)-0.56 A450. Lipid peroxidation was measured by using a method previously published by (Heath and Packer, 1968).

To estimate H_2_O_2_ content of plant tissues (root and leaf), 3 mL of sample extract was mixed with 1 mL of 0.1% titanium sulfate in 20% (v/v) H_2_SO_4_ and centrifuged at 6000 × g for 15 min. The yellow color intensity was evaluated at 410 nm. The H_2_O_2_ level was computed by extinction coefficient of 0.28 mmol^−1^ cm^−1^. The contents of H_2_O_2_ were measured by the method presented by (Jana and Choudhuri, 1981).

Stress-induced electrolyte leakage (EL) of uppermost stretched leaves was determined by using methodology of (Dionisio-Sese and Tobita, 1998). The leaves were cut into minor slices (5 mm length) and placed in test tubes having 8 mL distilled water. These tubes were incubated and transferred into water bath for 2 h prior to measuring the initial electrical conductivity (EC_1_). The samples were autoclaved at 121 °C for 20 min, and then cooled down to 25 °C before measuring the final electrical conductivity (EC_2_). Electrolyte leakage was calculated as by the following formula;

EL= (EC_1_/ EC_2_) × 100

### 2.5 Determination of antioxidant enzyme activities and relative gene expression

To evaluate enzyme activities, fresh leaves (0.5 g) were homogenised in liquid nitrogen and 5 mL of 50 mmol sodium phosphate buffer (pH 7.0) including 0.5 mmol EDTA and 0.15 mol NaCl. The homogenate was centrifuged at 12,000 × *g* for 10 min at 4 °C, and the supernatant was used for measurement of superoxidase dismutase (SOD) and peroxidase (POD) activities. SOD activity was assayed in 3 mL reaction mixture containing 50 mM sodium phosphate buffer (pH 7), 56 mM nitro blue tetrazolium, 1.17 mM riboflavin, 10 mM methionine and 100 μL enzyme extract. Finally, the sample was measured by using a spectrophotometer (xMark™ Microplate Absorbance Spectrophotometer; Bio-Rad). Enzyme activity was measured by using a method by (Chen and Pan, 1996) and expressed as U g^−1^ FW.

POD activity in the leaves was estimated by using the method of (Sakharov and Ardila, 1999) by using guaiacol as the substrate. A reaction mixture (3 mL) containing 0.05 mL of enzyme extract, 2.75 mL of 50 mM phosphate buffer (pH 7.0), 0.1 mL of 1% H_2_O_2_ and 0.1 mL of 4% guaiacol solution was prepared. Increases in the absorbance at 470 nm because of guaiacol oxidation was recorded for 2 min. One unit of enzyme activity was defined as the amount of the enzyme.

Catalase (CAT) activity was analyzed according to (Aebi, 1984). The assay mixture (3.0 mL) was comprised of 100 μL enzyme extract, 100 μL H_2_O_2_ (300 mM) and 2.8 mL 50 mM phosphate buffer with 2 mM ETDA (pH 7.0). The CAT activity was measured from the decline in absorbance at 240 nm as a result of H_2_O_2_ loss (*ε* = 39.4 mM^−1^ cm^−1^).

Ascorbate peroxidase (APX) activity was measured according to (Nakano and Asada, 1981). The mixture containing 100 μL enzyme extract, 100 μL ascorbate (7.5 mM), 100 μL H_2_O_2_ (300 mM) and 2.7 mL 25 mM potassium phosphate buffer with 2 mM EDTA (pH 7.0) was used for measuring APX activity. The oxidation pattern of ascorbate was estimated from the variations in wavelength at 290 nm (*ε* = 2.8 mM^−1^ cm^−1^).

Quantitative real-time PCR (RT-qPCR) assay was applied to investigate the expression levels of 4 stress-related genes, including Fe-SOD, POD, CAT and APX. Total RNA was extracted from leaf tissue samples using RNeasy Plant Mini kits (Qiagen, Manchester, UK). Contaminating DNA was then removed and first-strand cDNAs were prepared using Reverse Transcription kits (Qiagen, Manchester, UK). RT-qPCR analysis was conducted as reported in the protocol of QuantiTect SYBR Green PCR kit (Qiagen, Manchester, UK). Reaction volume and PCR amplification conditions were adjusted as mentioned by (El-Esawi et al., 2020). The gene amplifications of (Sirhindi et al., 2016) of the following genes are given in Table 2S.

### 2.6 Determination of non-enzymatic antioxidants, sugars and proline contents

Plant ethanol extracts were prepared for the determination of non-enzymatic antioxidants and some key osmolytes. For this purpose, 50 mg of plant dry material was homogenized with 10 mL ethanol (80%) and filtered through Whatman No. 41 filter paper. The residue was re-extracted with ethanol and the two extracts were pooled together to a final volume of 20 mL. The determination of ascorbic acid (Azuma et al., 1999), glutathione (Lewis et al., 1998) and total sugars (Dubois et al., 1956) was performed from the extracts.

Fresh leaf material (0.1 g) was mixed thoroughly in 5 mL aqueous sulphosalicylic acid (3%). The mixture was centrifuged at 10000 × g for 15 min and aliquot (1 mL) was poured into a test tube having 1 mL acidic ninhydrin and 1 mL glacial acetic acid. The reaction mixture was first heated at 100 °C for 10 min and then cooled in an ice bath. The reaction mixture was extracted with 4 mL toluene and test tubes are vortexed for 20 s and cooled. Thereafter, the light absorbance at 520 nm was measured by using UV–VIS spectrophotometer (Hitachi U-2910, Tokyo, Japan). The free proline content was determined on the basis of standard curve at 520 nm absorbance and expressed as µmol (g FW) ^−1^ (Bates et al., 1973).

### 2.7 Determination of nutrient contents

For nutrient analysis, plant roots and shoots were washed twice in redistilled water, dipped in 20 mM EDTA for 3 s and then, again washed with deionized water twice for the removal of adsorbed metal on plant surface. The washed samples were then oven dried for 24 h at 105 °C. The dried roots and shoots were digested by using wet digestion method in HNO_3_: HClO_4_ (7:3 V/V) until clear samples were obtained. Each sample was filtered and diluted with redistilled water up to 50 mL. The root and shoot contents of Na, K, and P and were analyzed by using Atomic Absorption Spectrophotometer (AAS) model Agilent 240FS-AA.

### 2.8 Statistical analysis

Statistical analysis of data was performed with analysis of variance (ANOVA) by using a statistical program Co-Stat version 6.2, Cohorts Software, 2003, Monterey, CA, USA. All the data obtained was tested by two-way analysis of variance (ANOVA). Thus, the differences between treatments were determined by using ANOVA, and the least significant difference test (*P* < 0.05) was used for multiple comparisons between treatment means. Logarithmic or inverse transformations were performed for data normalization, where necessary, prior to analysis. Pearson’s correlation analysis was performed to quantify relationships between various analysed variables. The graphical presentation was carried out by using Origin-Pro 2017. The Pearson correlation coefficients, heat-map analysis and principal component analysis between the measured variables of *H. vulgare* were also calculated using the RStudio software.

## 3 Results

### 3.1 Effect of different levels of salinity on plant growth and biomass under the foliar application of AsA

**Fig. 1:**
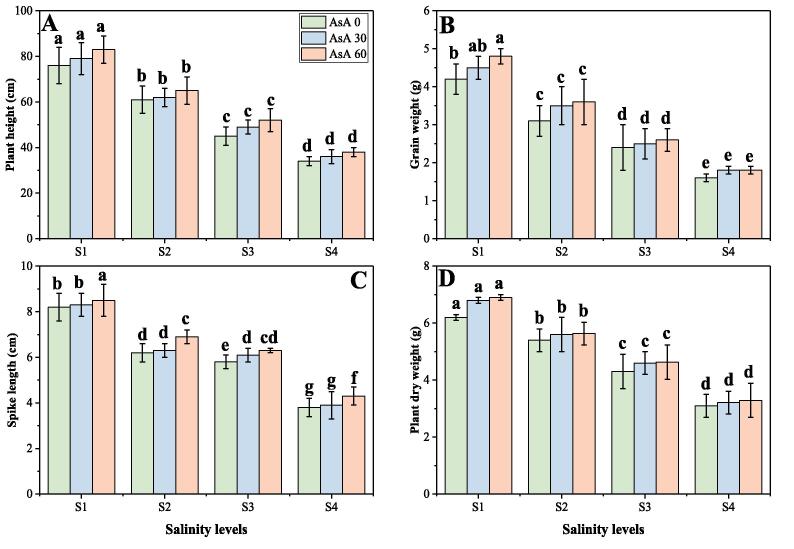
Effect of different concentrations of ascorbic acid on plant height (A), grain weight (B), plant fresh weight (C) and plant dry weight (D) under different levels of salinity on *H. vulgare*. Values are demonstrated as means of three replicates along with standard deviation (SD; n = 3). Two-way ANOVA was performed and means differences were tested by HSD (*p* < 0.05). Different lowercase letters on the error bars indicate significant difference between the treatments. Different levels of ascorbic acid application are as follow: AsA 0 (0 mM), AsA 30 (30 mM) and AsA 60 (60 mM). Different abbreviations of salinity are used as follow: S1 (0 mM), S2 (50 mM), S3 (100 mM) and S4 (150 mM).

In the present study, the effect of different saline levels (0, 50, 100 and 150 mM) on plant growth and biomass in *H. vulgare*, under different foliar levels of AsA (0, 30 and 60 mM) were also measured. The data regarding plant growth and biomass of *H. vulgare* are presented in Fig. 1. The findings from the present study are depicting that salinity stress induced a significant (*p* < 0.05) negative effect of plant growth and biomass in *H. vulgare*. Compared to the plants grown in 0 mM of NaCl in the soil, plant height, grain weight, plant fresh and dry biomass were decreased by 46.6, 64.2, 38.7 and 68.1% respectively in the plants grown in 150 mM of NaCl in the soil. However, results also showing that the plant growth and biomass were increased in saline soil by the application of AsA (Fig. 1). Results also showing that, plant height, grain weight, plant fresh and dry biomass were increased by 4.2, 5.4, 3.8 and 11.7% respectively in the plants grown in 150 mM of salinity in the soil with the foliar application of 60 mM of AsA, compared to those plants which were grown in 150 mM of salinity in the soil without the foliar application of of AsA.

### 3.2 Effect of different levels of salinity on photosynthetic pigments, gas exchange characteristics and stomatal properties under the foliar application of AsA

**Fig. 2:**
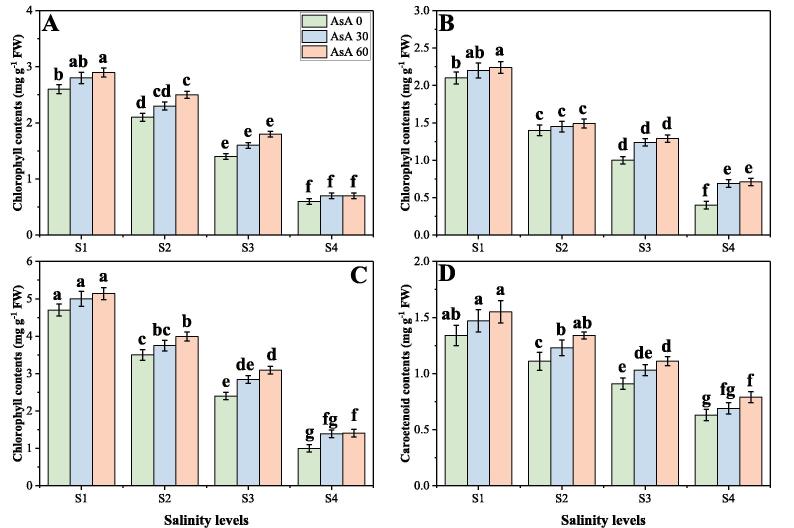
Effect of different concentrations of ascorbic acid on chlorophyll *a* contents (A), chlorophyll *b* contents (B), total chlorophyll contents (C) and carotenoid contents (D) under different levels of salinity on *H. vulgare*. Values are demonstrated as means of three replicates along with standard deviation (SD; n = 3). Two-way ANOVA was performed and means differences were tested by HSD (*p* < 0.05). Different lowercase letters on the error bars indicate significant difference between the treatments. Different levels of ascorbic acid application are as follow: AsA 0 (0 mM), AsA 30 (30 mM) and AsA 60 (60 mM). Different abbreviations of salinity are used as follow: S1 (0 mM), S2 (50 mM), S3 (100 mM) and S4 (150 mM).

**Fig. 3:**
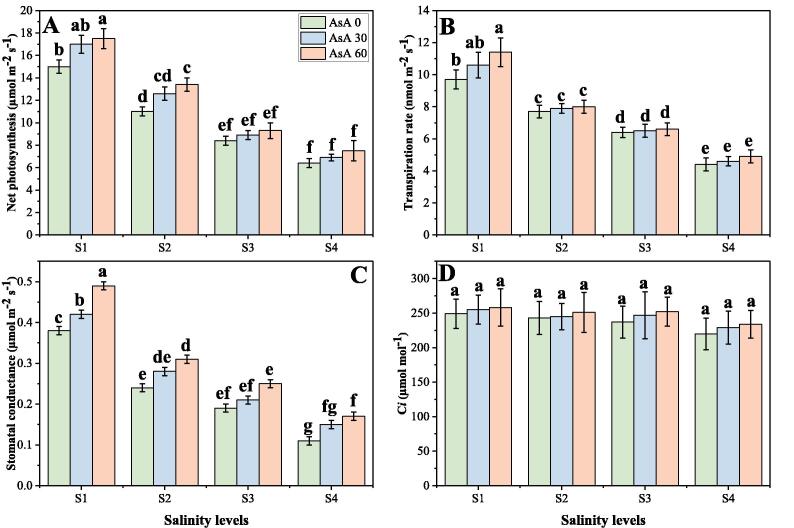
Effect of different concentrations of ascorbic acid on net photosynthesis (A), transpiration rate (B), stomatal conductance (C), and intercellular CO_2_ concentration (D) under different levels of salinity on *H. vulgare*. Values are demonstrated as means of three replicates along with standard deviation (SD; n = 3). Two-way ANOVA was performed and means differences were tested by HSD (*p* < 0.05). Different lowercase letters on the error bars indicate significant difference between the treatments. Different levels of ascorbic acid application are as follow: AsA 0 (0 mM), AsA 30 (30 mM) and AsA 60 (60 mM). Different abbreviations of salinity are used as follow: S1 (0 mM), S2 (50 mM), S3 (100 mM) and S4 (150 mM).

**Fig. 4:**
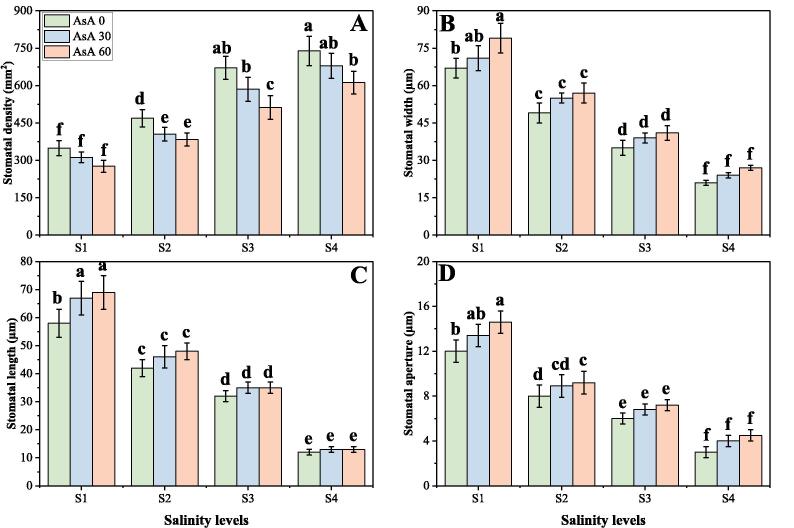
Effect of different concentrations of ascorbic acid on stomatal density (A), stomatal width (B), stomatal length (C) and stomatal aperture (D) under different levels of salinity on *H. vulgare*. Values are demonstrated as means of three replicates along with standard deviation (SD; n = 3). Two-way ANOVA was performed and means differences were tested by HSD (*p* < 0.05). Different lowercase letters on the error bars indicate significant difference between the treatments. Different levels of ascorbic acid application are as follow: AsA 0 (0 mM), AsA 30 (30 mM) and AsA 60 (60 mM). Different abbreviations of salinity are used as follow: S1 (0 mM), S2 (50 mM), S3 (100 mM) and S4 (150 mM).

In the present study, the photosynthetic pigments, gas exchange characteristics and stomatal properties were significantly (*p* < 0.05) decreased in the plants grown in the increasing levels of salinity in the soil, compared to those plants grown without the addition of salinity in the soil. Although, photosynthetic pigments, gas exchange characteristics and stomatal properties can be increased by the foliar application of AsA, when grown in elevating levels of salinity in the soil. The results regarding photosynthetic pigments, gas exchange parameters and stomatal properties in *H. vulgare* grown in different levels of salinity in the soil, with or without the application of AsA are presented in Fig. 2, Fig. 3, Fig. 4 respectively. Compared to the plants grown in 0 mM of NaCl in the soil, the total chlorophyll contents, carotenoid contents, net photosynthesis (P*n*), transpiration rate (T*r*), stomatal conductance (g*s*), stomatal width, stomatal length and stomatal aperture were decreased by 76.8, 56.0, 57.4, 51.2, 68.7, 84.6, 92.7 and 62.4% respectively in the plants grown in 150 mM of salt stress without the foliar application of AsA. We also advocated that intercellular CO_2_ (C*i*) was non-significant at all levels of salinity and AsA (Fig. 3D), while increasing levels of NaCl in the soil significantly (*p* < 0.05) increased stomatal density in *H. vulgare*, compared to those plants grown without addition of NaCl in the soil (Fig. 4A). Although, application of AsA increased chlorophyll contents, carotenoid contents, P*n*, T*r*, g*s*, stomatal width, stomatal length and stomatal aperture non-significantly by 28.7, 33.8, 12.8, 16.4, 28.7, 24.7, 6.4 and 16.7% respectively, at 150 mM of sodium chloride in the soil with the foliar supplied with 60 mM, compared with the plants grown in 150 mM of sodium chloride in the soil without the foliar application of AsA.

### 3.3 Effect of different levels of salinity on oxidative stress and enzymatic antioxidants under the foliar application of AsA

**Fig. 5:**
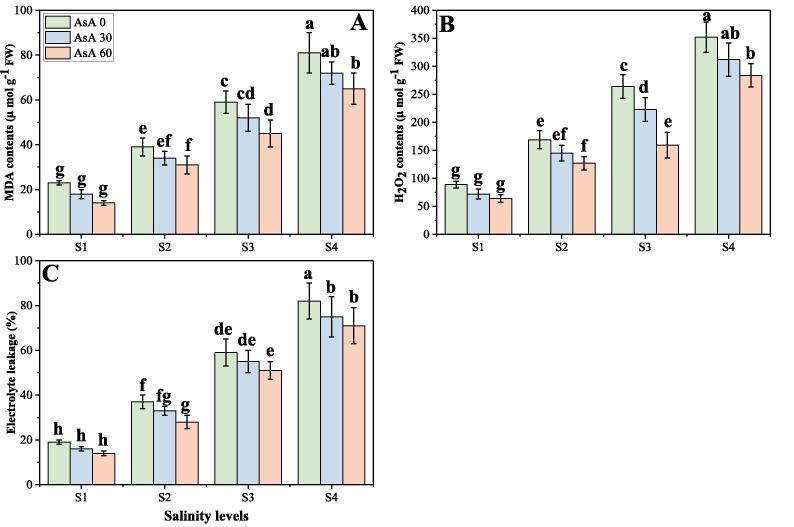
Effect of different concentrations of ascorbic acid on malondialdehyde (MDA) contents (A), H_2_O_2_ contents (B) and electrolyte leakage (C) under different levels of salinity on *H. vulgare*. Values are demonstrated as means of three replicates along with standard deviation (SD; n = 3). Two-way ANOVA was performed and means differences were tested by HSD (*p* < 0.05). Different lowercase letters on the error bars indicate significant difference between the treatments. Different levels of ascorbic acid application are as follow: AsA 0 (0 mM), AsA 30 (30 mM) and AsA 60 (60 mM). Different abbreviations of salinity are used as follow: S1 (0 mM), S2 (50 mM), S3 (100 mM) and S4 (150 mM).

**Fig. 6:**
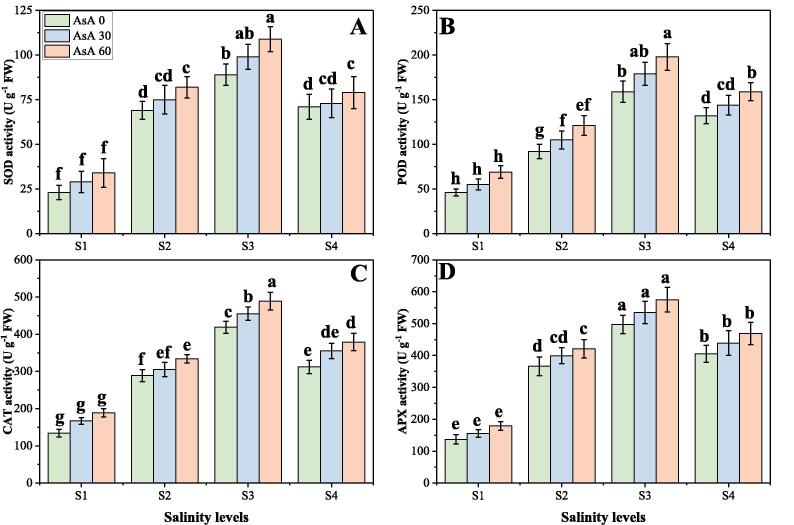
Effect of different concentrations of ascorbic acid on superoxidase dismutase (SOD) (A), peroxidase (POD) (B), catalase (CAT) (C) and ascorbate peroxidase (APX) (D) under different levels of salinity on *H. vulgare*. Values are demonstrated as means of three replicates along with standard deviation (SD; n = 3). Two-way ANOVA was performed and means differences were tested by HSD (*p* < 0.05). Different lowercase letters on the error bars indicate significant difference between the treatments. Different levels of ascorbic acid application are as follow: AsA 0 (0 mM), AsA 30 (30 mM) and AsA 60 (60 mM). Different abbreviations of salinity are used as follow: S1 (0 mM), S2 (50 mM), S3 (100 mM) and S4 (150 mM).

**Fig. 7:**
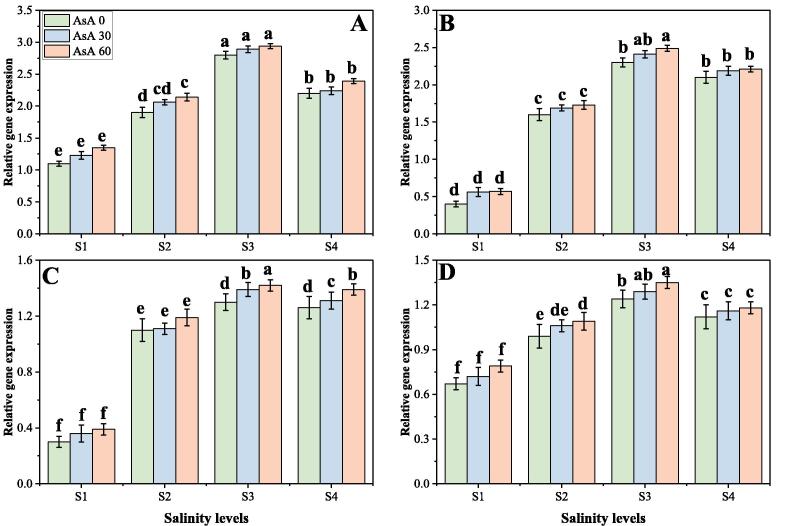
Effect of different concentrations of ascorbic acid of relative gene expression on superoxidase dismutase (Fe-SOD) (A), peroxidase (POD) (B), catalase (CAT) (C) and ascorbate peroxidase (APX) (D) under different levels of salinity on *H. vulgare*. Values are demonstrated as means of three replicates along with standard deviation (SD; n = 3). Two-way ANOVA was performed and means differences were tested by HSD (*p* < 0.05). Different lowercase letters on the error bars indicate significant difference between the treatments. Different levels of ascorbic acid application are as follow: AsA 0 (0 mM), AsA 30 (30 mM) and AsA 60 (60 mM). Different abbreviations of salinity are used as follow: S1 (0 mM), S2 (50 mM), S3 (100 mM) and S4 (150 mM).

In the present study, we also advocated various oxidative stress indicators, response of different enzymatic antioxidants and also their gene expression, under varying levels of salinity in the soil with or without the foliar application of AsA. The results regarding oxidative stress indicators, antioxidant enzymes and their relative gene expression are presented in Fig. 5, Fig. 6, Fig. 7 respectively, under increasing levels of NaCl in the soil with or without the foliar application of AsA. The findings from the present study advocated that the contents of malondialdehyde (MDA), hydrogen peroxide (H_2_O_2_) and electrolyte leakage [EL (%)] increased significantly (*p* < 0.05) under elevating concentrations of salinity (0, 50, 100 and 150 mM) in the soil, compared with the plants grown in non-saline soil (Fig. 5). The activities of various antioxidants (SOD, POD, CAT and APX) and relative gene expression (Fe-SOD, POD, CAT and APX) were increased initially up to a salinity level of 100 mM in the soil, while further addition of the NaCl in the soil (150 mM) caused a significant (*p* < 0.05) decreased in the activities of antioxidants and relative gene expression. These results also advocated that application of AsA significantly (*p* < 0.05) decreased oxidative stress indicators (Fig. 5), while significantly (*p* < 0.05) increased activities of antioxidant enzymes (Fig. 6), while non-significantly (*p* < 0.05) increased expression of their related genes (Fig. 7), compared to those plants which were grown without the foliar application of AsA. According to the given results, the contents of MDA, H_2_O_2_, EL (%) and were increased by 287.5, 241.2 and 327.0% respectively in the plants grown in 150 mM of sodium chloride, compared with 0 mM of sodium chloride in the soil (Fig. 5). Compared to the plants grown in non-saline soil activities of antioxidants such as SOD, POD, CAT and APX were increased by 180.4, 284.5, 347.6 and 371.0% respectively (*p* < 0.05) and their relative gene expression such as Fe-SOD, POD, CAT and APX were increased by 166.6, 246.4, 160.4 and 69.7% respectively in the plants grown in 100 mM of NaCl in the soil, compared to those plants grown without saline soil. Although, the foliar application of AsA non-significantly (*p* < 0.05) decreased the contents of MDA, H_2_O_2_, EL (%) by 12.4, 39.7 and 16.7% respectively at 150 mM of NaCl in the soil, compared to those plants grown at 0 mM of NaCl in the soil (Fig. 5). At all salinity levels, the foliar application of AsA significantly (*p* < 0.05) increased activities of antioxidants and their relative gene expression compared to those plants which grown without the foliar application of AsA (Fig. 6, Fig. 7).

### 3.4 Effect of different levels of salinity on non-enzymatic compound and osmolytes under the foliar application of AsA

**Fig. 8:**
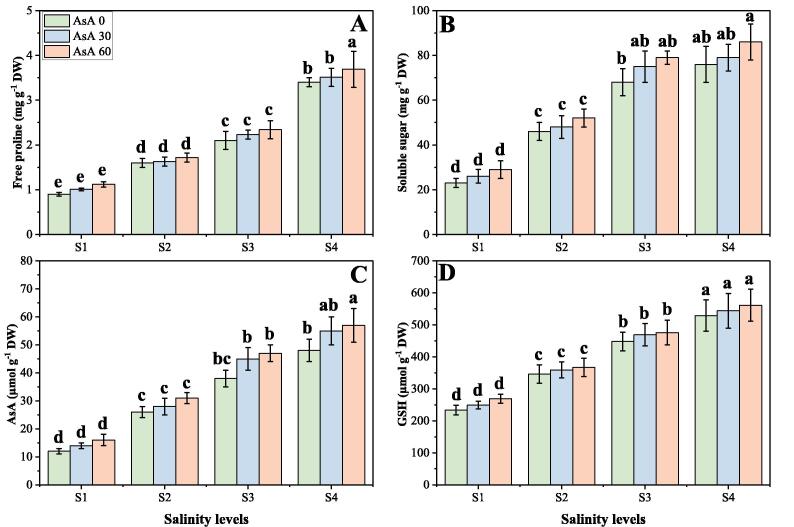
Effect of different concentrations of ascorbic acid on free proline (A), soluble sugar (B), ascorbic acid (C) and Glutathione contents (D) under different levels of salinity on *H. vulgare*. Values are demonstrated as means of three replicates along with standard deviation (SD; n = 3). Two-way ANOVA was performed and means differences were tested by HSD (*p* < 0.05). Different lowercase letters on the error bars indicate significant difference between the treatments. Different levels of ascorbic acid application are as follow: AsA 0 (0 mM), AsA 30 (30 mM) and AsA 60 (60 mM). Different abbreviations of salinity are used as follow: S1 (0 mM), S2 (50 mM), S3 (100 mM) and S4 (150 mM).

The results regarding non-enzymatic antioxidants [AsA and Glutathione (GSH) contents] and osmolytes (free proline and soluble sugar) are presented in Fig. 8. These results are showing that increasing levels of salinity in the soil cause a significant (*p* < 0.05) increase in non-enzymatic compounds and osmolytes, compared to those plants which grown in non-saline soil (Fig. 8). Results also showing that the contents of AsA, GSH, free proline and soluble sugar were increased by 449.7, 263.3, 384.6 and 549.6% respectively at saline level of 150 mM in the soil, compared to those plants which grown without the addition of NaCl in the soil. We also elucidated that application of AsA further increased the contents of AsA, GSH, free proline and soluble sugar non-significantly (*p* < 0.05) by 8.4, 6.1, 16.8 and 7.5% respectively at saline level of 150 mM with the foliar application of AsA (60 mM), compared to those plants which were grown at saline level of 150 mM without the foliar application of AsA (Fig. 8).

### 3.5 Effect of different levels of salinity on ion uptake under the foliar application of AsA

**Fig. 9:**
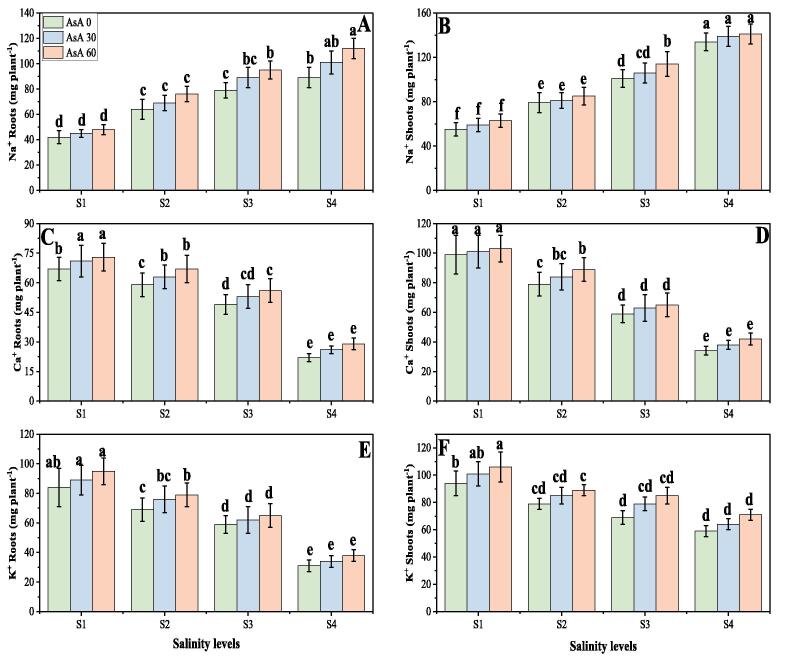
Effect of different concentrations of ascorbic acid on sodium content in roots (A), sodium content in shoots (B), calcium content in roots (C), calcium content in shoots (D), potassium content in roots (E) and potassium content in shoots (F) under different levels of salinity on *H. vulgare*. Values are demonstrated as means of three replicates along with standard deviation (SD; n = 3). Two-way ANOVA was performed and means differences were tested by HSD (*P* < 0.05). Different lowercase letters on the error bars indicate significant difference between the treatments. Different levels of ascorbic acid application are as follow: AsA 0 (0 mM), AsA 30 (30 mM) and AsA 60 (60 mM). Different abbreviations of salinity are used as follow: S1 (0 mM), S2 (50 mM), S3 (100 mM) and S4 (150 mM).

In the present study, different nutrients such as sodium (Na^+^), calcium (Ca^2+^) and potassium (K^+^) were also determined from roots and shoots of *H. vulgare* grown in varying levels of salinity with or without the application of AsA. The ion uptake from roots and shoots of *H. vulgare* grown in various levels of salinity with or without the application of AsA are presented in Fig. 9. According to the results, we have advocated that increasing concentration of salinity in the soil (0, 50, 100 and 150 mM) significantly (*p* < 0.05) increased Na^+^ while decreased Ca^2+^ and K^+^ in both (roots and shoots) parts of the plants, compared with the plants grown in non-saline soil (Fig. 9). We further elucidated that the foliar application of AsA non-significantly (*p* < 0.05) increased the contents of these ions in roots and shoots of the plants, compared with the plants grown without application of AsA at all levels of salinity (Fig. 9). Compared to the plants grown in non-saline soil, the contents of Na^+^ increased by 167.5% while contents of Ca^2+^ and K^+^ were decreased by 84.6 and 64.2% respectively in the roots and also the contents of Na^+^ increased by 203.2% while contents of Ca^2+^ and K^+^ were decreased by 26.4 and 19.7% respectively in the shoots were detected in the plants grown in 150 mM of NaCl in the soil. The contents of Na^+^, Ca^2+^ and K^+^ were increased by foliar application of AsA, which were incremented by 6.7, 5.6, 11.2% respectively in the roots and also increased by 3.4, 5.9 and 8.9% respectively in the shoots at 150 mM of NaCl in the soil with the foliar application of AsA (60 mM), compared to those plants which were grown in 150 mM of sodium chloride in the soil without the foliar application of AsA (0 mM).

### 3.6 Relationship between growth, physiological attributes and ions uptake in H. Vulgare

**Fig. 10:**
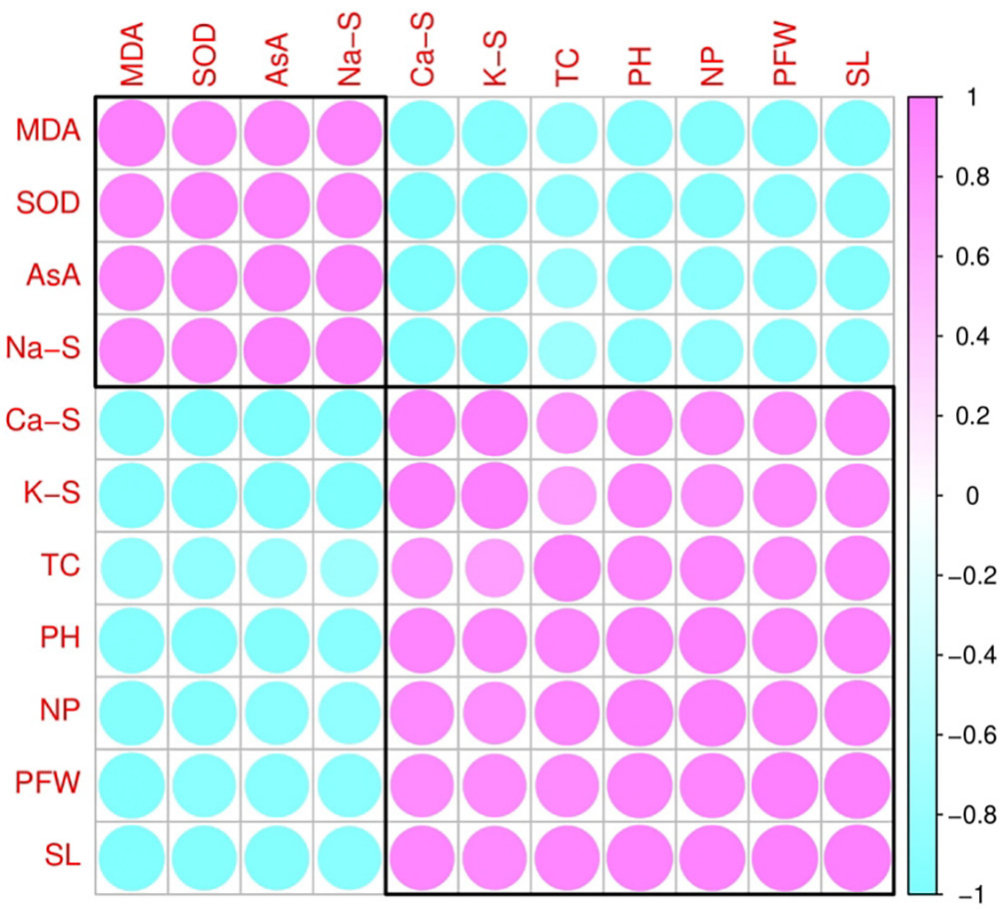
Correlation between different morphological and physiological traits of *H. vulgare* studied in this experiment. The abbreviations are as follows: SOD (superoxidase activity in the leaves), K-S (potassium content in shoots), MDA (malondialdehyde content in leaves), Na-S (sodium content in shoots), AsA (ascorbic acid contents in the leaves), Ca-S (calcium content in the shoots), TC (total chlorophyll contents), PH (plant height), NP (net photosynthesis), PFW (plant fresh weight) and SL (stomatal length).

**Fig. 11:**
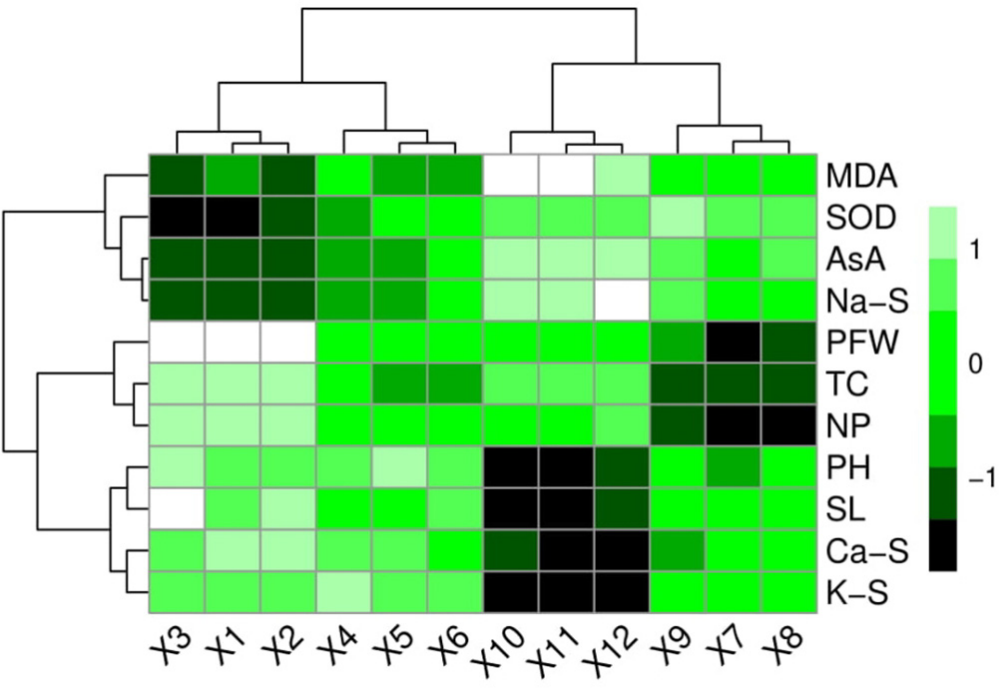
Heatmap histogram correlation between different morphological and physiological traits of *H. vulgare* studied in this experiment. The abbreviations are as follows: SOD (superoxidase activity in the leaves), K-S (potassium content in shoots), MDA (malondialdehyde content in leaves), Na-S (sodium content in shoots), AsA (ascorbic acid contents in the leaves), Ca-S (calcium content in the shoots), TC (total chlorophyll contents), PH (plant height), NP (net photosynthesis), PFW (plant fresh weight) and SL (stomatal length). The different treatments are represented as (X1) (salinity 0 mM + AsA 0 mM), (X2) (salinity 0 mM + AsA 30 mM), (X3) (salinity 0 mM + AsA 60 mM), (X4) (salinity 50 mM + AsA 0 mM), (X5) (salinity 50 mM + AsA 30 mM), (X6) (salinity 50 mM + AsA 60 mM), (X7) (salinity 100 mM + AsA 0 mM), (X8) (salinity 100 mM + AsA 30 mM), (X9) (salinity 100 mM + AsA 60 mM), (X10) (salinity 150 mM + AsA 0 mM), (X11) (salinity 150 mM + AsA 30 mM) and (X12) (salinity 150 mM + AsA 60 mM).

The Pearson correlation analysis was carried out to quantify the relationship between different studied parameters of *H. vulgare* grown in the saline soil with or without the application of AsA (Fig. 10). Na+ uptake in the shoots was negatively correlated with K+ and Ca2+ uptake in the shoots and positively correlated with MDA and AsA contents and activity of SOD. However, Ca2+ uptake by the shoots was positively correlated with K+ up take by the shoots but negatively correlated with MDA and AsA contents and activity of SOD. However, K+ up taken by the shoots was also negatively correlated with the contents of MDA and AsA and the activity of SOD and positively correlated with total chlorophyll contents, plant height, net photosynthesis, plant fresh weight and stomatal length. This correlation depicted a close connection between ion uptake by the plants with different morpho-physio-biochemical attributes of H. vulgare grown in various concentrations of salinity with or without the application AsA. A heat-map histogram was also illustrated between variable of various treatments studied in this experiment (Fig. 11). These variables showing the similar results as showed in the correlation analysis (Fig. 11).

### 3.7 Principal component analysis

**Fig. 12:**
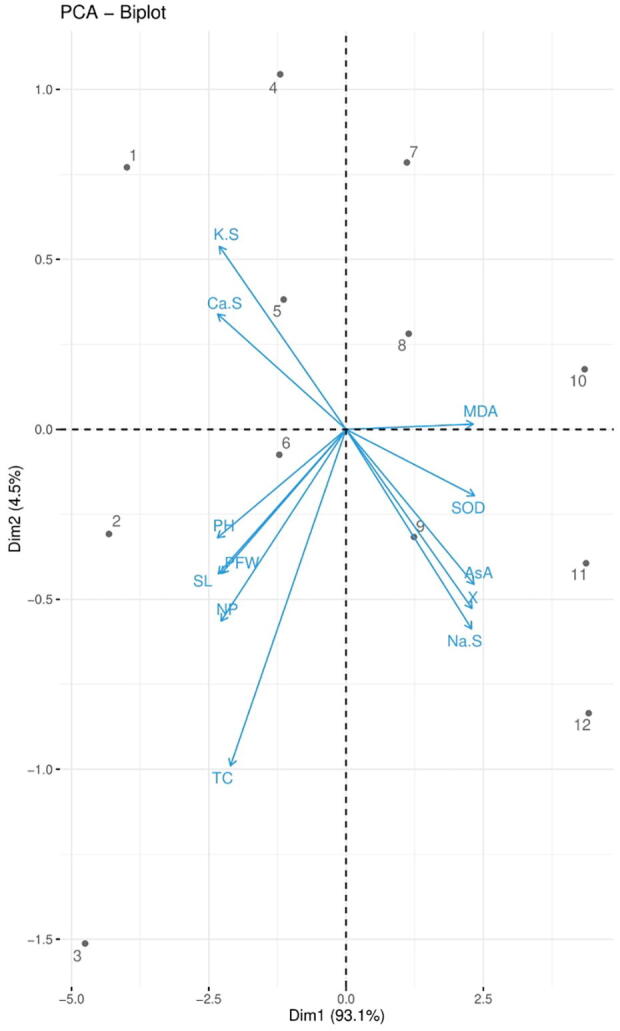
Score and loading plot of principal component analysis (PCA) on different morphological and physiological traits of *H. vulgare* under different levels of salinity with or without the application of AsA. Score plot represents separation of treatments as (1) (salinity 0 mM + AsA 0 mM), (2) (salinity 0 mM + AsA 30 mM), (3) (salinity 0 mM + AsA 60 mM), (4) (salinity 50 mM + AsA 0 mM), (5) (salinity 50 mM + AsA 30 mM), (6) (salinity 50 mM + AsA 60 mM), (7) (salinity 100 mM + AsA 0 mM), (8) (salinity 100 mM + AsA 30 mM), (9) (salinity 100 mM + AsA 60 mM), (10) (salinity 150 mM + AsA 0 mM), (11) (salinity 150 mM + AsA 30 mM) and (12) (salinity 150 mM + AsA 60 mM). The abbreviations are as follows: SOD (superoxidase activity in the leaves), K-S (potassium content in shoots), MDA (malondialdehyde content in leaves), Na-S (sodium content in shoots), AsA (ascorbic acid contents in the leaves), Ca-S (calcium content in the shoots), TC (total chlorophyll contents), PH (plant height), NP (net photosynthesis), PFW (plant fresh weight) and SL (stomatal length).

The scores and loading plots of PCA to evaluate the effect of different levels of salinity with or without the foliar application of AsA in H. vulgare are presented in Fig. 12. Of all the main components, the first two components-Dim1 and Dim2-comprise more than 97% of the whole database and make up the largest portion of all components. Among this, Dim1 contributes 93.1%, and Dim2 contributes 4.5% of the whole dataset. According to the results, all the respected treatments were dispersed successfully in the whole dataset. The distribution of all the components in the dataset gives a clear indication that the salt stress significantly affected different morpho-physio-biochemical attributes at all the treatments studied in this experiment, with or without the foliar application of AsA. The treatment (3) was most displaced from all other treatments of the present study, indicating that the positive role of AsA in plant growth and physiology of *H. vulgare* grown with or without the addition of sodium chloride in the soil. However, PCA also showing that Na^+^ uptake in the shoots and also MDA and AsA contents and activity of SOD were positively correlated with all other respected treatments in the database. In contrast, K+ and Ca2+ uptake in the shoots, total chlorophyll contents, plant height, net photosynthesis, plant fresh weight and stomatal length were negatively correlated in database.

## 4 Discussion

For two reasons, salts in soil water can inhibit plant growth. Firstly, the presence of salt in the soil solution decreases the water absorption capacity of the plant and this contributes to a reduction in the rate of growth. This is referred to as salinity's osmotic or water-deficit effect. Secondly, if excessive amounts of salt enter the plant in the transpiration stream, there will be injury to cells in the transpiring leaves and this may cause further reductions in growth. This is called the saline-specific or ion-excess salinity effect (Chantre Nongpiur et al., 2016, Kang et al., 2014, Munns and Tester, 2008). Furthermore, as the death of plants and/or declines in productivity, the adverse effects of high salinity on plants can be observed at the entire plant stage. All essential processes such as plant growth and biomass, photosynthesis, protein synthesis, and energy and lipid metabolism are impaired during the onset and production of salt stress within a plant (Adhikari et al., 2020, Safdar et al., 2019, Zafar et al., 2015). Three-fold effects of salt stress; viz. It decreases water potential and induces ion imbalance or ion homeostasis and toxicity disturbances. This altered status of water leads to an initial decrease in growth and a decline in plant productivity. Since both osmotic and ionic stress are involved in salt stress (Adhikari et al., 2020, Chantre Nongpiur et al., 2016). Previously, high concentration of salinity in the soil significantly (*p* < 0.05) decreased plant growth and biomass in *Sisyrinchium angustofolium* (Nizam, 2011), *H. vulgare* (Xu et al., 2012), *Cucumis sativus* (Kang et al., 2014) and *Brassica oleracea* (Hassini et al., 2017). In the present study, we also advocated that increasing saline levels in the soil (0, 50, 100 and 150 mM) caused a significant (*p* < 0.05) decreased in plant growth and biomass in *H. vulgare*, compared to those plants which grown in 0 mM of NaCl in the soil (Fig. 1). Salinity stress results in a clear stunting of plants (Jing et al., 2018, Parida and Das, 2005, Zafar et al., 2015), because of the lower osmotic capacity of the germination media, it induces toxicity that alters the activities of nucleic acid metabolism enzymes, alters protein metabolism, disturbs hormonal equilibrium, and decreases the use of seed reserves (Adhikari et al., 2020, Jabeen and Ahmad, 2017, Kang et al., 2014, Xu et al., 2012, Zafar et al., 2015).

In general, the chlorophyll and carotenoid content of leaves decreases under salt stress. With extended periods of salt stress, the oldest leaves begin to develop chlorosis and fall (Chantre Nongpiur et al., 2016, Dawood and El-Awadi, 2015, Saleem et al., 2020b, Saleem et al., 2020g). However, the decrease in gaseous exchange parameters in plants under salt stress is primarily due to the decrease in the demand for water. Photosynthesis is also inhibited when large Na^+^ and/or Cl^-^ concentrations are accumulated in the chloroplasts, and chlorophyll is a major photosynthesis material that directly correlates to plant development (Chantre Nongpiur et al., 2016, Dolatabadian et al., 2011, Jabeen and Ahmad, 2017). High concentrations of salt in soil and water create a high osmotic potential that decreases plant water supply. Osmotic stress is caused by a decrease in water potential, which reversibly inactivates photosynthetic electron transport by intercellular space shrinkage (Dolatabadian et al., 2011, Parihar et al., 2015, Sadak and Abdelhamid, 2015). In the present study, we also advocated that salinity treatments were associated with the reduction in photosynthetic pigments (Fig. 2), gas exchange characteristics (Fig. 3) and stomatal properties (Fig. 4), which is similar to previously described (Dolatabadian et al., 2011, Shah et al., 2011). The reduction of CO_2_ supply because of closure of stomata. The reduction in stomatal conductance results in limited availability of CO_2_ for carboxylation reactions (Dolatabadian and Jouneghani, 2009, Hassini et al., 2017, Kamran et al., 2019). Interestingly, the efficiency of photosystem II (PSII) in the tolerant *Triticum aestivum* accession was unaffected, while there was a decline in the quantum yield of PSII photochemistry, coinciding with leaf ageing, higher Na^+^ and/or Cl^−^ concentrations in the leaf, and chlorophyll degradation, in the sensitive genotype (James et al., 2002). The internal reduction of CO_2_ reduces the activity of many enzymes, including RuBisCo, following stomatal closure, thus restricting carboxylation and reducing the net photosynthetic rate. The intercellular CO_2_ concentration (C*i*) is another parameter that has been used to estimate the effects of salinity on photosynthesis. In the saline stress environment, the CO_2_ assimilation rate (as a function of C*i*) was shown to be better maintained by a salt tolerant species, of *Aribdopsis* (Stepien and Johnson, 2009). However, it is difficult to differentiate cause–effect relationships between photosynthesis (source) and growth reduction (sink); also, the effects of salinity on photosynthesis can be caused by alterations in the photosynthetic metabolism, or else by secondary effects caused by oxidative stress (Ali et al., 2020, Xu et al., 2012).

Besides the direct influence of salinity on plants, a common consequence of salinity is induction of excessive accumulation of reactive oxygen species (ROS) which can cause peroxidation of lipids, oxidation of protein, inactivation of enzymes, DNA alteration, and/or interact with other important constituents of plant cells (Kala, 2015, Shafiq et al., 2020). Soil salinity can lead to stomatal closure, which reduces CO_2_ availability in the leaves and inhibits carbon fixation, exposing chloroplasts to excessive excitation energy which in turn increase the production of ROS such as superoxide (O_2_^•–^), hydrogen peroxide (H_2_O_2_), hydroxyl radical (OH^•^), and singlet oxygen (^1^O_2_) (Alam et al., 2020, Ali et al., 2020, Islam et al., 2016). The reactive oxygen species that are by-products of hyperosmotic and ionic stresses cause membrane disfunction and causes cell death (Imran et al., 2020, Mohamed et al., 2020, Parveen et al., 2020, Rana et al., 2020, Rehman et al., 2020, Saleem et al., 2020i, Shahid et al., 2020, Yaseen et al., 2020). The plants defend against these ROS by induction of activities of varieties of antioxidative enzymes such as superoxide dismutase (SOD), peroxidase (POD) and catalase (CAT) and ascorbate peroxidase (APX), which scavenge ROS (Hameed et al., 2021, Imran et al., 2020, Saleem et al., 2020c, Saleem et al., 2020d, Saleem et al., 2020e). Besides activation of ROS scavenging enzymes, other responses could be observed in salt-treated plants. An increased abundance of tocopherol cyclase, a crucial enzyme in the biosynthesis of α-tocopherol, an important compound in plant non-enzymatic ROS scavenging mechanisms, and also increased in free proline, GSH and AsA has been found in Chinese halophytic plant *Puccinellia tenuiflora* (Yu et al., 2011). Previous studies have been shown that soil salinity increases activities of antioxidant compounds (enzymatic and non-enzymatic) in *H. vulgare* (Zhou et al., 2012), *Cucumis sativus* (Kang et al., 2014) and *Vicia faba* (Sadak and Abdelhamid, 2015) grown in saline soil or sea water saline soil. Moreover, abiotic-stress-related genes also increases to mitigates anxious effect of salinity stress which was previously reported by Barakat (Barakat, 2003). The increasing levels of salinity caused a significant (*p* < 0.05) increase in oxidative stress indicators (Fig. 5), enzymatic antioxidant compounds (enzymatic and non-enzymatic) (Fig. 6, Fig. 8) and their relative gene expression (Fig. 7), when compared to those plants grown in non-saline soil. The activities of antioxidants initially increased up to a saline level of 100 mM in the soil, then further increment in soil salinity cause a significant (*p* < 0.05) decrease in the activities of antioxidant enzymes. The decrease in antioxidant activities at saline level of 100 mM is directly associated with their relative gene expression (Fig. 7) and also function of some enzymes essential for protein biosynthesis (Kamran et al., 2019).

Ion uptake is important not only for normal development, but also for plant growth in saline environment, as stress disrupts ion homeostasis (Liu et al., 2020). The nutritional disorders can arise from the impact of salinity on the availability, competitive absorption, transport or distribution of nutrients within the plant. Numerous studies have shown that salinity decreases nutrient absorption and nutrient accumulation in the plants (Dawood and El-Awadi, 2015, Tsamaidi et al., 2017). The availability of micronutrients in saline soils depends on the solubility of micronutrients, soil solution pH, soil solution redox potential, and the presence of binding sites on the surfaces of organic and inorganic particles. In addition, depending on crop species and salinity levels, salinity can influence micronutrient concentrations in plants differently (Kang et al., 2014, Sadak and Abdelhamid, 2015, Safdar et al., 2019). In the present study, we also advocated that increasing soil salinity caused a significant (*p* < 0.05) increase in Na^+^, up taken by the roots and shoots in *H. vulgare* (Fig. 9A, B), which can reduce plant growth and found to be similar findings associated with (Liu et al., 2020) in *H. vulgare*. We also found that salinity stress causes a significant (*p* < 0.05) decline in the contents of K^+^ and Ca^2+^ up taken by the roots and shoots in *H. vulgare* (Fig. 9C, D, E and F). It was also reported that high NaCl in the soil causes a significant reduction in the contents of K^+^ and Ca^2+^ and may lead to plant death (Gupta and Huang, 2014, Parihar et al., 2015).

Ascorbate or ascorbic acid (AsA) is the most abundant antioxidant found in plants. It participates in the redox system of the plant by serving as an electron donor to different reactions (Bilska et al., 2019, Khan et al., 2011, Ullah et al., 2016). Moreover, AsA (also known as “vitamin C”) plays a central role in plant defence, which is widely used in cell metabolism and is necessary to synthesize hydroxyproline containing proteins (Al-Hakimi and HAMAdA, 2011, Munir and Aftab, 2011). However, as a result of a complex sequence of biochemical reactions such as activation or suppression of main enzymatic reactions, induction of synthesis of stress-responsive proteins and the development of different chemical protection compounds, it regulates stress response (Dolatabadian and Jouneghani, 2009, Khan et al., 2011, Zhou et al., 2016). Although the growing growth of plants and biomass under AsA application may accelerate cell division and cell enlargement and improve the integrity of the membrane, which may have led to reducing ion leakage and thus improving growth (Akram et al., 2017). Increasing of net photosynthesis and in transpiration rate indicated that these vitamins probably reflect the efficiency of water uptake and utilization or reduction of water loss, which consequently causes increase in leaf water potential. Hence, it could be concluded that the beneficial effect of vitamins on growth parameters of *H. vulgare* plants has been related to the efficiency of their water uptake and utilization (Fig. 1, Fig. 2). This increase in plant growth could attribute with the physiological processes including nutrient uptake, photosynthesis and increasing antioxidant activities as suggested by (Alhasnawi et al., 2015). Our results also showed that the negative impact of soil salinity can overcome by the foliar application of AsA and can increase photosynthetic pigments (Fig. 2), gas exchange characteristics (Fig. 3) and stomatal properties (Fig. 4) in *H. vulgare* at all levels of salinity stress. These findings are coincide by the findings of (Azooz et al., 2012), when they studied *Vicia faba* under various levels of salinity and noticed that application of vitamins increased photosynthetic pigments and gas exchange parameters. They suggested that increasing contents of photosynthetic pigments under the treatments of AsA, might be due to the protecting role of these vitamins. Researchers also reported that high contents of salinity in the soil may lead to increase ROS in the chloroplast of the cell and which causes the distortion of chlorophyll molecules, while vitamin C can detoxify/nullify the negative effect of these ROS free radicles, which may lead to increase in chlorophyll contents and decrease in oxidative stress in the plants (Alhasnawi et al., 2015, Farooq et al., 2013, Sharma et al., 2019). It was also reported that foliar application of AsA mitigates adverse effect of soil salinity on plant growth and composition by increasing leaf area and photosynthetic pigments in the plant (Azooz et al., 2012, Munné-Bosch and Alegre, 2002).

The imbalance in the production and removal of ROS is the key threat to the plant cell in the environment of soil salinity, but additional ROS formation is also thought to serve as a signaling molecule to the active plant defense system (Saleem et al., 2019a, Saleem et al., 2019b). We have depicted that soil salinity increased antioxidant activities (enzymatic and non-enzymatic) (Fig. 6, Fig. 8) and relative gene expression (Fig. 7), associated with the increase in oxidative stress in the plants (Fig. 5). Meanwhile, the activities of antioxidant compounds further increased with the application of AsA which indicates that soil salinity induced stress tolerance in *H. vulgare* which in turn reduce the contents of MDA, H_2_O_2_ and EL (%) (Fig. 5). The increase in antioxidants in salinity stress and also with the exogenous application of AsA might be due to signaling molecule causing up-regulation of relative gene expression and specific factors related with the antioxidant activities (Al-Hakimi and HAMAdA, 2011, Aziz et al., 2018, Fatima et al., 2019). These results indicate the key role of AsA in eliminating toxic free radicals and conferring salinity stress tolerance through upregulating the abiotic stress-related genes. It is important to mention that application of AsA increased the contents of Na ^+^, K^+^ and Ca^2+^ in roots and shoots of *H. vulgare* at all levels of salinity in the soil (Fig. 9). It was reported in number of studies that application of AsA being a non-enzymatic antioxidant and as a plant growth regular usually stimulates nutrients uptake in different plant species (Younis et al., 2010, Zhou et al., 2016) and diminish toxic effect of soil salinity (Dolatabadian and Jouneghani, 2009). Though, it was also reported that application of AsA decrease the Na^+^ contents in the plants, but up-regulates K^+^ and Ca^2+^ contents in salt stressed soil (Alhasnawi et al., 2015, Dolatabadian and Jouneghani, 2009, Munir and Aftab, 2011); so, exogenous application of AsA could be a potential plant growth regulator, which increase the plant resistance against saline condition.

## 5 Conclusion

The results of this experiment confirm that soil salinity causes a significant adverse effect on plant growth and biomass, photosynthetic pigments, gas exchange characteristics, stomatal properties, ion uptake and exclusion imbalance, and also causes ROS production and its elimination within the cell. Furthermore, plant has strong defense system which scavenged ROS production and also increasing activities of antioxidants has been influenced by the relative gene expression study through RT-qPCR. Although, *H. vulgare* is believed to be a salinity tolerant cereal crop, but to enhance plant growth and composition we also used AsA application which not only increase plant growth and biomass, but also booted up photosynthetic machinery, plant defense system and up taken of essential nutrients from the soil. However, AsA is its self in non-enzymatic antioxidant compound and decreases oxidative stress in the plant by elimination of ROS generation. Hence, foliar application of AsA mitigates salinity stress in *H. vulgare* and can be used as a plant growth regulator under the saline environment. Further studies need to be conducted to find the possible mechanism on molecular level in cereal crops, under saline environment.

## 6 Ethics approval

Not Applicable

## 7 Consent to participate

All authors consent to participate in this manuscript Consent for publication

All authors consent to publish this manuscript in Saudi journal of Biological Science

## 8 Availability of data and material

Data will be available on request to the corresponding or first author

## 9 Code availability

Not Applicable

All authors have read the paper and satisfied with the final manuscript.

## CRediT authorship contribution statement

**Amara Hassan:** Conceptualization, Formal analysis, Resources, Supervision, Funding acquisition. **Syeda Fasiha Amjad:** Conceptualization, Software, Visualization. **Muhammad Hamzah Saleem:** Methodology, Validation, Data curation, Writing - original draft. **Humaira Yasmin:** Conceptualization, Software, Data curation, Writing - review & editing, Project administration, Funding acquisition. **Muhammad Imran:** Conceptualization, Software, Data curation, Data curation, Project administration, Funding acquisition. **Muhammad Riaz:** Conceptualization, Methodology, Validation, Investigation, Writing - review & editing, Funding acquisition. **Qurban Ali:** Methodology, Formal analysis, Writing - original draft, Visualization. **Faiz Ahmad Joyia:** Methodology, Data curation, Writing - review & editing, Project administration, Funding acquisition. **Mobeen:** Methodology, Validation, Data curation, Writing - original draft, Writing - review & editing, Visualization. **Shakeel Ahmed:** Software, Formal analysis, Writing - original draft, Writing - review & editing, Project administration. **Shafaqat Ali:** Writing - review & editing, Supervision, Funding acquisition. **Abdulaziz Abdullah Alsahli:** Writing - original draft, Writing - review & editing, Supervision, Project administration, Funding acquisition. **Mohammed Nasser Alyemeni:** Writing - original draft, Writing - review & editing, Supervision, Funding acquisition.

## Declaration of Competing Interest

The authors declare that they have no known competing financial interests or personal relationships that could have appeared to influence the work reported in this paper.

## References

[b0005] Abdel-Hamid A.M., Mohamed H.I. (2014). The effect of the exogenous gibberellic acid on two salt stressed barley cultivars. European Sci. J..

[b0010] Adhikari B., Dhungana S.K., Kim I.-D., Shin D.-H. (2020). Effect of foliar application of potassium fertilizers on soybean plants under salinity stress. J. Saudi Soc. Agri. Sci..

[b0015] Aebi H. (1984). Catalase in vitro. Meth. Enzymol. Elsevier.

[b0020] Agami R. (2014). Applications of ascorbic acid or proline increase resistance to salt stress in barley seedlings. Biol. Plant..

[b0025] Akhlaghi H., Mahdavi B., Rezaei H. (2018). Characterization of Chemical Composition and Antioxidant Properties of Trachyspermum ammi Seed as a Potential Medicinal Plant. J. Chem. Health Risks.

[b0030] Akram N.A., Shafiq F., Ashraf M. (2017). Ascorbic acid-a potential oxidant scavenger and its role in plant development and abiotic stress tolerance. Front. Plant Sci..

[b0035] Al-Hakimi, A.-B., HAMAdA, A.M., 2011. Ascorbic acid, thiamine or salicylic acid induced changes in some physiological parameters in wheat grown under copper stress. Plant Protection Science 47, 92-108.

[b0040] Alam H., Khattak J.Z.K., Ksiksi T.S., Saleem M.H., Fahad S., Sohail H., Ali Q., Zamin M., El-Esawi M.A., Saud S. (2020). Negative impact of long-term exposure of salinity and drought stress on native Tetraena mandavillei L. Physiol. Plant..

[b0045] Alhasnawi A.N., Kadhimi A.A., Isahak A., Mohamad A., Yusoff W.M.W., Zain C.R.C.M. (2015). Exogenous application of ascorbic acid ameliorates detrimental effects of salt stress in rice (MRQ74 and MR269) seedlings. Asian J. Crop Sci..

[b0050] Ali M., Kamran M., Abbasi G.H., Saleem M.H., Ahmad S., Parveen A., Malik Z., Afzal S., Ahmar S., Dawar K.M., Ali S., Alamri S., Siddiqui M.H., Akbar R., Fahad S. (2020). Melatonin-Induced Salinity Tolerance by Ameliorating Osmotic and Oxidative Stress in the Seedlings of Two Tomato (Solanum lycopersicum L.) Cultivars. J. Plant Growth Regul..

[b0055] Ali, Q., Ahmar, S., Sohail, M.A., Kamran, M., Ali, M., Saleem, M.H., Rizwan, M., Ahmed, A.M., Mora-Poblete, F., do Amaral Júnior, A.T., 2021. Research advances and applications of biosensing technology for the diagnosis of pathogens in sustainable agriculture. Environmental Science and Pollution Research, 1-18. 10.1007/s11356-021-12419-6 33464530

[b0060] Ali S., Zeng F., Qiu L., Zhang G. (2011). The effect of chromium and aluminum on growth, root morphology, photosynthetic parameters and transpiration of the two barley cultivars. Biol. Plant..

[b0065] Aly A.A., Khafaga A.F., Omar G.N. (2012). Improvement the adverse effect of salt stress in Egyptian clover (Trifolium alexandrinum L.) by AsA application through some biochemical and RT-PCR markers. J. Appl. Phytotechnol. Environ. Sanitation.

[b0070] Arnon D.I. (1949). Copper enzymes in isolated chloroplasts. Polyphenoloxidase in Beta vulgaris. Plant Physiol..

[b0075] Austin, R.B., 1990. Prospects for genetically increasing the photosynthetic capacity of crops.

[b0080] Aziz A., Akram N.A., Ashraf M. (2018). Influence of natural and synthetic vitamin C (ascorbic acid) on primary and secondary metabolites and associated metabolism in quinoa (Chenopodium quinoa Willd.) plants under water deficit regimes. Plant Physiol. Biochem..

[b0085] Azooz M.M., Abou-Elhamd M.F., Al-Fredan M.A. (2012). Biphasic effect of copper on growth, proline, lipid peroxidation and antioxidant enzyme activities of wheat ('Triticum aestivum'cv. Hasaawi) at early growing stage. Aust. J. Crop Sci..

[b0090] Azuma K., Nakayama M., Koshioka M., Ippoushi K., Yamaguchi Y., Kohata K., Yamauchi Y., Ito H., Higashio H. (1999). Phenolic antioxidants from the leaves of Corchorus olitorius L. J. Agric. Food. Chem..

[b0095] Barakat H. (2003). Interactive effects of salinity and certain vitamins on gene expression and cell division. Int. J. Agri. Biol..

[b0100] Bates L.S., Waldren R.P., Teare I. (1973). Rapid determination of free proline for water-stress studies. Plant Soil.

[b0105] Bilska K., Wojciechowska N., Alipour S., Kalemba E.M. (2019). Ascorbic Acid—The Little-Known Antioxidant in Woody Plants. Antioxidants.

[b0110] Bolat I., Kaya C., Almaca A., Timucin S. (2006). Calcium sulfate improves salinity tolerance in rootstocks of plum. J. Plant Nutr..

[b0115] Chantre Nongpiur R., Lata Singla-Pareek S., Pareek A. (2016). Genomics approaches for improving salinity stress tolerance in crop plants. Curr. Genomics.

[b0120] Chen C.-N., Pan S.-M. (1996). Assay of superoxide dismutase activity by combining electrophoresis and densitometry. Botanical Bullet. Academia Sinica.

[b0125] Dawood M.G., El-Awadi M.E. (2015). Alleviation of salinity stress on Vicia faba L. plants via seed priming with melatonin. Acta Biológica Colombiana.

[b0130] Deng G., Yang M., Saleem M.H., Rehman M., Fahad S., Yang Y., Elshikh M.S., Alkahtani J., Ali S., Khan S.M. (2021). Nitrogen fertilizer ameliorate the remedial capacity of industrial hemp (Cannabis sativa L.) grown in lead contaminated soil. J. Plant Nutr..

[b0135] Dionisio-Sese M.L., Tobita S. (1998). Antioxidant responses of rice seedlings to salinity stress. Plant Sci..

[b0140] Dolatabadian A., Jouneghani R.S. (2009). Impact of exogenous ascorbic acid on antioxidant activity and some physiological traits of common bean subjected to salinity stress. Notulae Botanicae Horti Agrobotanici Cluj-Napoca.

[b0145] Dolatabadian, A., SANAVY, S.A.M.M., Ghanati, F., 2011. Effect of salinity on growth, xylem structure and anatomical characteristics of soybean. Notulae Scientia Biologicae 3, 41-45.

[b0150] Dubois, M., Gilles, K.A., Hamilton, J.K., Rebers, P.t., Smith, F., 1956. Colorimetric method for determination of sugars and related substances. Analytical chemistry 28, 350-356.

[b0155] El-Esawi M.A., Elkelish A., Soliman M., Elansary H.O., Zaid A., Wani S.H. (2020). Serratia marcescens BM1 enhances cadmium stress tolerance and phytoremediation potential of soybean through modulation of osmolytes, leaf gas exchange, antioxidant machinery, and stress-responsive genes expression. Antioxidants.

[b0160] Farooq M., Irfan M., Aziz T., Ahmad I., Cheema S. (2013). Seed priming with ascorbic acid improves drought resistance of wheat. J. Agron. Crop Sci..

[b0165] Fatima A., Singh A.A., Mukherjee A., Agrawal M., Agrawal S.B. (2019). Ascorbic acid and thiols as potential biomarkers of ozone tolerance in tropical wheat cultivars. Ecotoxicol. Environ. Saf..

[b0170] Gupta B., Huang B. (2014). Mechanism of salinity tolerance in plants: physiological, biochemical, and molecular characterization. Int. Jo. Genomics 2014.

[b0175] Gupta N., Thind S.K. (2017). Grain yield response of drought stressed wheat to foliar application of glycine betaine. Indian J. Agri. Res..

[b0180] Hameed A., Akram N.A., Saleem M.H., Ashraf M., Ahmed S., Ali S., Abdullah Alsahli A., Alyemeni M.N. (2021). Seed Treatment with α-Tocopherol Regulates Growth and Key Physio-Biochemical Attributes in Carrot (Daucus carota L.) Plants under Water Limited Regimes. Agronomy.

[b0185] Hashmat S., Shahid M., Tanwir K., Abbas S., Ali Q., Niazi N.K., Akram M.S., Saleem M.H., Javed M.T. (2021). Elucidating distinct oxidative stress management, nutrient acquisition and yield responses of Pisum sativum L. fertigated with diluted and treated wastewater. Agric. Water Manag..

[b0190] Hassini I., Martinez-Ballesta M.C., Boughanmi N., Moreno D.A., Carvajal M. (2017). Improvement of broccoli sprouts (Brassica oleracea L. var. italica) growth and quality by KCl seed priming and methyl jasmonate under salinity stress. Sci. Hortic..

[b0195] Heath R.L., Packer L. (1968). Photoperoxidation in isolated chloroplasts: I. Kinetics and stoichiometry of fatty acid peroxidation. Arch. Biochem. Biophys..

[b0200] Hossain B., Akhtar M. (2014). Growth and yield of barley (Hordeum vulgare L.) as affected by irrigation, sowing method and phosphorus level. Academia J. Agri. Res..

[b0205] Imran M., Hussain S., El-Esawi M.A., Rana M.S., Saleem M.H., Riaz M., Ashraf U., Potcho M.P., Duan M., Rajput I.A. (2020). Molybdenum Supply Alleviates the Cadmium Toxicity in Fragrant Rice by Modulating Oxidative Stress and Antioxidant Gene Expression. Biomolecules.

[b0210] Iqbal S., Khan M.M., Ahmad R., Ahmed W., Tahir T., Jaskani M.J., Ahmed S., Iqbal Q., Hussnain R. (2015). Morpho-physiological and biochemical response of citrus rootstocks to salinity stress at early growth stage. Pak. J. Agri. Sci.

[b0215] Islam F., Yasmeen T., Arif M.S., Ali S., Ali B., Hameed S., Zhou W. (2016). Plant growth promoting bacteria confer salt tolerance in Vigna radiata by up-regulating antioxidant defense and biological soil fertility. Plant Growth Regul..

[b0220] Jabeen N., Ahmad R. (2017). Growth response and nitrogen metabolism of sunflower (Helianthus annuus L.) to vermicompost and biogas slurry under salinity stress. J. Plant Nutr..

[b0225] James R.A., Rivelli A.R., Munns R., von Caemmerer S. (2002). Factors affecting CO2 assimilation, leaf injury and growth in salt-stressed durum wheat. Funct. Plant Biol..

[b0230] Jana S., Choudhuri M.A. (1981). Glycolate metabolism of three submersed aquatic angiosperms: effect of heavy metals. Aquat. Bot..

[b0235] Javed, M.T., Saleem, M.H., Aslam, S., Rehman, M., Iqbal, N., Begum, R., Ali, S., Alsahli, A.A., Alyemeni, M.N., Wijaya, L., 2020. Elucidating silicon-mediated distinct morpho-physio-biochemical attributes and organic acid exudation patterns of cadmium stressed Ajwain (Trachyspermum ammi L.). Plant Physiology and Biochemistry. 10.1016/j.plaphy.2020.10.010 33069978

[b0240] Javed, M.T., Tanwir, K., Abbas, S., Saleem, M.H., Iqbal, R., Chaudhary, H.J., 2021. Chromium retention potential of two contrasting Solanum lycopersicum Mill. cultivars as deciphered by altered pH dynamics, growth, and organic acid exudation under Cr stress. Environmental Science and Pollution Research, 1-13. 10.1007/s11356-020-12269-8 33511536

[b0245] Jiang X., Li H., Song X. (2016). Seed priming with melatonin effects on seed germination and seedling growth in maize under salinity stress. Pak J Bot.

[b0250] Jing X., Yang J., Wang T. (2018). Effects of salinity on herbicide lactofen residues in soil. Water Air Soil Pollut..

[b0255] Kala S. (2015). Effect of Nacl salt stress on antioxidant enzymes of isabgol (Plantago ovata Forsk.) Genotypes. Int. J. Food Sci. Tech. Res.

[b0260] Kamran M., Parveen A., Ahmar S., Malik Z., Hussain S., Chattha M.S., Saleem M.H., Adil M., Heidari P., Chen J.-T. (2019). An Overview of Hazardous Impacts of Soil Salinity in Crops, Tolerance Mechanisms, and Amelioration through Selenium Supplementation. Int. J. Mol. Sci..

[b0265] Kang S.-M., Khan A.L., Waqas M., You Y.-H., Kim J.-H., Kim J.-G., Hamayun M., Lee I.-J. (2014). Plant growth-promoting rhizobacteria reduce adverse effects of salinity and osmotic stress by regulating phytohormones and antioxidants in Cucumis sativus. J. Plant Interact..

[b0270] Kaya C., Ashraf M., Sonmez O., Tuna A.L., Polat T., Aydemir S. (2015). Exogenous application of thiamin promotes growth and antioxidative defense system at initial phases of development in salt-stressed plants of two maize cultivars differing in salinity tolerance. Acta Physiolog. Plantarum.

[b0275] Khan, M.M., Al-Mas’oudi, R.S., Al-Said, F., Khan, I., 2013. Salinity effects on growth, electrolyte leakage, chlorophyll content and lipid peroxidation in cucumber (Cucumis sativus L.), International Conference on Food and Agricultural Sciences Malaysia: IACSIT Press, pp. 28-32.

[b0280] Khan T., Mazid M., Mohammad F. (2011). A review of ascorbic acid potentialities against oxidative stress induced in plants. Journal of Agrobiology.

[b0285] Lewis C.E., Walker J.R., Lancaster J.E., Sutton K.H. (1998). Determination of anthocyanins, flavonoids and phenolic acids in potatoes. I: Coloured cultivars of Solanum tuberosum L. J. Sci. Food Agric..

[b0290] Li P., Zhu Y., Song X., Song F. (2020). Negative effects of long-term moderate salinity and short-term drought stress on the photosynthetic performance of Hybrid Pennisetum. Plant Physiol. Biochem..

[b0295] Liu L., Nakamura Y., Taliman N.A., Sabagh A.E., Moghaieb R.E., Saneoka H. (2020). Differences in the Growth and Physiological Responses of the Leaves of Peucedanum japonicum and Hordeum vulgare Exposed to Salinity. Agriculture.

[b0300] Mohamed, I.A., Shalby, N., MA El-Badri, A., Saleem, M.H., Khan, M.N., Nawaz, M.A., Qin, M., Agami, R.A., Kuai, J., Wang, B., 2020. Stomata and Xylem Vessels Traits Improved by Melatonin Application Contribute to Enhancing Salt Tolerance and Fatty Acid Composition of Brassica napus L. Plants. Agronomy 10, 1186.

[b0305] Munir N., Aftab F. (2011). Enhancement of salt tolerance in sugarcane by ascorbic acid pretreatment. Afr. J. Biotechnol..

[b0310] Munné-Bosch S., Alegre L. (2002). Interplay between ascorbic acid and lipophilic antioxidant defences in chloroplasts of water-stressed Arabidopsis plants. FEBS Lett..

[b0315] Munns R., Tester M. (2008). Mechanisms of salinity tolerance. Annu. Rev. Plant Biol..

[b0320] Nakano Y., Asada K. (1981). Hydrogen peroxide is scavenged by ascorbate-specific peroxidase in spinach chloroplasts. Plant Cell Physiol..

[b0325] Nazar Z., Akram N.A., Saleem M.H., Ashraf M., Ahmed S., Ali S., Abdullah Alsahli A., Alyemeni M.N. (2020). Glycinebetaine-Induced Alteration in Gaseous Exchange Capacity and Osmoprotective Phenomena in Safflower (Carthamus tinctorius L.) under Water Deficit Conditions. Sustainability.

[b0330] Nizam I. (2011). Effects of salinity stress on water uptake, germination and early seedling growth of perennial ryegrass. Afr. J. Biotechnol..

[b0335] Parida A.K., Das A.B. (2005). Salt tolerance and salinity effects on plants: a review. Ecotoxicol. Environ. Saf..

[b0340] Parihar P., Singh S., Singh R., Singh V.P., Prasad S.M. (2015). Effect of salinity stress on plants and its tolerance strategies: a review. Environ. Sci. Pollut. Res..

[b0345] Parveen A., Saleem M.H., Kamran M., Haider M.Z., Chen J.-T., Malik Z., Rana M.S., Hassan A., Hur G., Javed M.T. (2020). Effect of Citric Acid on Growth, Ecophysiology, Chloroplast Ultrastructure, and Phytoremediation Potential of Jute (Corchorus capsularis L.) Seedlings Exposed to Copper Stress. Biomolecules.

[b0350] Rahneshan Z., Nasibi F., Moghadam A.A. (2018). Effects of salinity stress on some growth, physiological, biochemical parameters and nutrients in two pistachio (Pistacia vera L.) rootstocks. J. Plant Interact..

[b0355] Rana M.S., Hu C.X., Shaaban M., Imran M., Afzal J., Moussa M.G., Elyamine A.M., Bhantana P., Saleem M.H., Syaifudin M. (2020). Soil phosphorus transformation characteristics in response to molybdenum supply in leguminous crops. J. Environ. Manage..

[b0360] REHMAN, M., FAHAD, S., SALEEM, M.H., HAFEEZ, M., RAHMAN, M.H., LIU, F., DENG, G., 2020. Red light optimized physiological traits and enhanced the growth of ramie (Boehmeria nivea L.). Photosynthetica.

[b0365] Rehman M., Liu L., Bashir S., Saleem M.H., Chen C., Peng D., Siddique K.H. (2019). Influence of rice straw biochar on growth, antioxidant capacity and copper uptake in ramie (Boehmeria nivea L.) grown as forage in aged copper-contaminated soil. Plant Physiol. Biochem..

[b0370] Sadak M.S., Abdelhamid M.T. (2015). Influence of amino acids mixture application on some biochemical aspects, antioxidant enzymes and endogenous polyamines of Vicia faba plant grown under seawater salinity stress. Gesunde Pflanzen.

[b0375] Safdar H., Amin A., Shafiq Y., Ali A., Yasin R., Shoukat A., Hussan M.U., Sarwar M.I. (2019). A review: impact of salinity on plant growth. Nat Sci.

[b0380] Sajid Z.A., Aftab F. (2009). Amelioration of salinity tolerance in Solanum tuberosum L. by exogenous application of ascorbic acid. Vitro Cellular Developmental Biol.-Plant.

[b0385] Sakharov I.Y., Ardila G.B. (1999). Variations of peroxidase activity in cocoa (Theobroma cacao L.) beans during their ripening, fermentation and drying. Food Chem..

[b0390] Saleem M., Ali S., Rehman M., Rana M., Rizwan M., Kamran M., Imran M., Riaz M., Hussein M., Elkelish A., Lijun L. (2020). Influence of phosphorus on copper phytoextraction via modulating cellular organelles in two jute (Corchorus capsularis L.) varieties grown in a copper mining soil of Hubei Province, China. Chemosphere.

[b0395] Saleem M.H., Ali S., Hussain S., Kamran M., Chattha M.S., Ahmad S., Aqeel M., Rizwan M., Aljarba N.H., Alkahtani S. (2020). Flax (Linum usitatissimum L.): A Potential Candidate for Phytoremediation? Biological and Economical Points of View. Plants.

[b0400] Saleem, M.H., Ali, S., Irshad, S., Hussaan, M., Rizwan, M., Rana, M.S., Hashem, A., Abd_Allah, E.F., Ahmad, P., 2020c. Copper Uptake and Accumulation, Ultra-Structural Alteration, and Bast Fibre Yield and Quality of Fibrous Jute (Corchorus capsularis L.) Plants Grown Under Two Different Soils of China. Plants 9, 404. 10.3390/plants9030404 PMC7154872 32213938

[b0405] Saleem M.H., Ali S., Kamran M., Iqbal N., Azeem M., Tariq Javed M., Ali Q., Zulqurnain Haider M., Irshad S., Rizwan M. (2020). Ethylenediaminetetraacetic Acid (EDTA) Mitigates the Toxic Effect of Excessive Copper Concentrations on Growth, Gaseous Exchange and Chloroplast Ultrastructure of Corchorus capsularis L. and Improves Copper Accumulation Capabilities. Plants.

[b0410] Saleem M.H., Ali S., Seleiman M.F., Rizwan M., Rehman M., Akram N.A., Liu L., Alotaibi M., Al-Ashkar I., Mubushar M. (2019). Assessing the Correlations between Different Traits in Copper-Sensitive and Copper-Resistant Varieties of Jute (Corchorus capsularis L.). Plants.

[b0415] Saleem M.H., Fahad S., Adnan M., Ali M., Rana M.S., Kamran M., Ali Q., Hashem I.A., Bhantana P., Ali M., Hussain R.M. (2020). Foliar application of gibberellic acid endorsed phytoextraction of copper and alleviates oxidative stress in jute (Corchorus capsularis L.) plant grown in highly copper-contaminated soil of China.

[b0420] Saleem M.H., Fahad S., Khan S.U., Ahmar S., Khan M.H.U., Rehman M., Maqbool Z., Liu L. (2020). Morpho-physiological traits, gaseous exchange attributes, and phytoremediation potential of jute (Corchorus capsularis L.) grown in different concentrations of copper-contaminated soil. Ecotoxicol. Environ. Saf..

[b0425] Saleem M.H., Fahad S., Rehman M., Saud S., Jamal Y., Khan S., Liu L. (2020). Morpho-physiological traits, biochemical response and phytoextraction potential of short-term copper stress on kenaf (Hibiscus cannabinus L.) seedlings. PeerJ.

[b0430] Saleem M.H., Kamran M., Zhou Y., Parveen A., Rehman M., Ahmar S., Malik Z., Mustafa A., Anjum R.M.A., Wang B. (2020). Appraising growth, oxidative stress and copper phytoextraction potential of flax (Linum usitatissimum L.) grown in soil differentially spiked with copper. J. Environ. Manage..

[b0435] Saleem M.H., Rehman M., Kamran M., Afzal J., Noushahi H.A., Liu L. (2020). Investigating the potential of different jute varieties for phytoremediation of copper-contaminated soil. Environ. Sci. Pollut. Res..

[b0440] Saleem M.H., Rehman M., Zahid M., Imran M., Xiang W., Liu L. (2019). Morphological changes and antioxidative capacity of jute (Corchorus capsularis, Malvaceae) under different color light-emitting diodes. Brazilian. J. Botany.

[b0445] Shafiq F., Iqbal M., Ashraf M.A., Ali M. (2020). Foliar applied fullerol differentially improves salt tolerance in wheat through ion compartmentalization, osmotic adjustments and regulation of enzymatic antioxidants. Physiol. Mol. Biol. Plants.

[b0450] Shah, S.S., Mohammad, F., Shafi, M., BAKHT, J., ZHOU, W., 2011. Effects of cadmium and salinity on growth and photosynthesis parameters of Brassica species. Pakistan Journal of Botany 43, 333-340.

[b0455] Shahid, M., Javed, M.T., Tanwir, K., Akram, M.S., Tazeen, S.K., Saleem, M.H., Masood, S., Mujtaba, S., Chaudhary, H.J., 2020. Plant growth-promoting Bacillus sp. strain SDA-4 confers Cd tolerance by physio-biochemical improvements, better nutrient acquisition and diminished Cd uptake in Spinacia oleracea L. Physiology and Molecular Biology of Plants, 1-17. 10.1007/s12298-020-00900-4 PMC7772128 33424156

[b0460] Sharma R., Bhardwaj R., Thukral A.K., Al-Huqail A.A., Siddiqui M.H., Ahmad P. (2019). Oxidative stress mitigation and initiation of antioxidant and osmoprotectant responses mediated by ascorbic acid in Brassica juncea L. subjected to copper (II) stress. Ecotoxicol. Environ. Saf..

[b0465] Sirhindi G., Mir M.A., Abd-Allah E.F., Ahmad P., Gucel S. (2016). Jasmonic acid modulates the physio-biochemical attributes, antioxidant enzyme activity, and gene expression in Glycine max under nickel toxicity. Front. Plant Sci..

[b0470] Stepien P., Johnson G.N. (2009). Contrasting responses of photosynthesis to salt stress in the glycophyte Arabidopsis and the halophyte Thellungiella: role of the plastid terminal oxidase as an alternative electron sink. Plant Physiol..

[b0475] Tsamaidi D., Daferera D., Karapanos I., Passam H. (2017). The effect of water deficiency and salinity on the growth and quality of fresh dill (Anethum graveolens L.) during autumn and spring cultivation. International Journal of Plant. Production.

[b0480] Ullah H.A., Javed F., Wahid A., Sadia B. (2016). Alleviating effect of exogenous application of ascorbic acid on growth and mineral nutrients in cadmium stressed barley (Hordeum vulgare) seedlings. Int. J. Agri. Biol..

[b0485] Wu D., Cai S., Chen M., Ye L., Chen Z., Zhang H., Dai F., Wu F., Zhang G. (2013). Tissue metabolic responses to salt stress in wild and cultivated barley. PLoS ONE.

[b0490] Xu, R., Wang, J., Li, C., Johnson, P., Lu, C., Zhou, M., 2012. A single locus is responsible for salinity tolerance in a Chinese landrace barley (Hordeum vulgare L.). PloS one 7. 10.1371/journal.pone.0043079 PMC3423432 22916210

[b0495] Yaseen R., Aziz O., Saleem M.H., Riaz M., Zafar-ul-Hye M., Rehman M., Ali S., Rizwan M., Nasser Alyemeni M., El-Serehy H.A. (2020). Ameliorating the Drought Stress for Wheat Growth through Application of ACC-Deaminase Containing Rhizobacteria along with Biogas Slurry. Sustainability.

[b0500] Younis M.E., Hasaneen M.N., Kazamel A.M. (2010). Exogenously applied ascorbic acid ameliorates detrimental effects of NaCl and mannitol stress in Vicia faba seedlings. Protoplasma.

[b0505] Yu J., Chen S., Zhao Q., Wang T., Yang C., Diaz C., Sun G., Dai S. (2011). Physiological and proteomic analysis of salinity tolerance in Puccinellia tenuiflora. J. Proteome Res..

[b0510] Zafar S., Ashraf M.Y., Niaz M., Kausar A., Hussain J. (2015). Evaluation of wheat genotypes for salinity tolerance using physiological indices as screening tool. Pak. J. Bot.

[b0515] Zaheer, I.E., Ali, S., Saleem, M.H., Ali, M., Riaz, M., Javed, S., Sehar, A., Abbas, Z., Rizwan, M., El-Sheikh, M.A., Alyemeni, M.N., 2020a. Interactive role of zinc and iron lysine on Spinacia oleracea L. growth, photosynthesis and antioxidant capacity irrigated with tannery wastewater. Physiology and Molecular Biology of Plants. 10.1007/s12298-020-00912-0 PMC7772129 33424157

[b0520] Zaheer I.E., Ali S., Saleem M.H., Arslan Ashraf M., Ali Q., Abbas Z., Rizwan M., El-Sheikh M.A., Alyemeni M.N., Wijaya L. (2020). Zinc-lysine Supplementation Mitigates Oxidative Stress in Rapeseed (Brassica napus L.) by Preventing Phytotoxicity of Chromium, When Irrigated with Tannery Wastewater. Plants.

[b0525] Zaheer I.E., Ali S., Saleem M.H., Imran M., Alnusairi G.S.H., Alharbi B.M., Riaz M., Abbas Z., Rizwan M., Soliman M.H. (2020). Role of iron–lysine on morpho-physiological traits and combating chromium toxicity in rapeseed (Brassica napus L.) plants irrigated with different levels of tannery wastewater. Plant Physiol. Biochem..

[b0530] Zaheer I.E., Ali S., Saleem M.H., Noor I., El-Esawi M.A., Hayat K., Rizwan M., Abbas Z., El-Sheikh M.A., Alyemeni M.N. (2020). Iron-Lysine Mediated Alleviation of Chromium Toxicity in Spinach (Spinacia oleracea L.) Plants in Relation to Morpho-Physiological Traits and Iron Uptake When Irrigated with Tannery Wastewater. Sustainability.

[b0535] Zamin M., Khattak A.M. (2017). Performance of Sporobolus spicatus ecotypes, UAE native grass, under various salinity levels. Pure and Applied Biology (PAB).

[b0540] Zhou G., Johnson P., Ryan P.R., Delhaize E., Zhou M. (2012). Quantitative trait loci for salinity tolerance in barley (Hordeum vulgare L.). Mol. Breed..

[b0545] Zhou X., Gu Z., Xu H., Chen L., Tao G., Yu Y., Li K. (2016). The effects of exogenous ascorbic acid on the mechanism of physiological and biochemical responses to nitrate uptake in two rice cultivars (Oryza sativa L.) under aluminum stress. J. Plant Growth Regul..

