# Supplementary material for: Foliar application of ascorbic acid enhances salinity stress tolerance in barley (Hordeum vulgare L.) through modulation of morpho-physio-biochemical attributes, ions uptake, osmo-protectants and stress response genes expression
Source: Saudi J Biol Sci. 2021 Mar 21;28(8):4276–90. doi: 10.1016/j.sjbs.2021.03.045 (PMC8324950; doi:10.1016/j.sjbs.2021.03.045)
Supplement: Supplementary data 1 [file mmc1.docx]

**Table 1S** Physic-chemical properties of non-saline soil used in the pot experiment

| **Texture** | **Clay loam** |
| --- | --- |
| pH | 6.9 |
| Electrical conductivity (EC) (dS cm^−1^) | 0.9 |
| Cation exchange capacity (CEC) (cmol kg^−1^) 4.9 | 4.78 |
| Soluble CO3^-2^ (mmol L^−1^) | 0.85 |
| Soluble HCO_3_ (mmol L^−1^) | 3.45 |
| Soluble Cl^−^ (mmol L^−1^) | 2.3 |
| Soluble Ca^2+^ + Mg^2+^ (mmol L^−1^) | 14.93 |
| Organic matter (g kg^−1^) | 71 |
| P (g kg^−1^) | 0.17 |
| K (g kg^−1^) | 21 |
| N (g kg^−1^) | 16 |
|  |  |

| Gene | Primer Sequence (5′-3′) | Gene Accession Number | Reference |
| --- | --- | --- | --- |
| Fe-SOD | F: ATCTTAGTTATGGTTCTCTTTGT  R: ATGGTGTAGAGCCTTTTCATAT | M64267 | [47] |
| POD | F: TTGAAATAAAC CAAAGGAGTAGT  R: AATAATTATTTGAATCTCTTTAAGG | AF145349 | [47] |
| CAT | F: AGCATCTCACCTGAACTTGAA  R: AGGTGAGAGGTTTGTGGCC | AF035252 | [47] |
| APX | F: CGTGACGATGATTGGGAAGT  R: TGATAGTGATCTTTCGGACCT | NM_001354113 | [47] |

**Table 2S** Gene-specific primers sequences used in the present study
